# CMV titer associations with cognition and the plasma proteome implicate FLT1 and neurovascular mechanisms as potential moderators

**DOI:** 10.1186/s12974-026-03800-8

**Published:** 2026-04-16

**Authors:** Michael R. Duggan, Erin Jacobsen, Shuojia Yang, Xuemei Zeng, Sridhar Kandala, Cassandra M. Joynes, Thomas K. Karikari, Vishwajit L. Nimgaonkar, M. Ilyas Kamboh, Beth E. Snitz, Luigi Ferrucci, Robert Yolken, Mary Ganguli, Keenan A. Walker

**Affiliations:** 1 Laboratory of Behavioral Neuroscience, National Institute On Aging, National Institutes of Health, NIH Biomedical Research Center Bg. RM. 04B316, 251 Bayview Blvd, Baltimore, MD 21224 USA; 2 Department of Psychiatry, University of Pittsburgh School of Medicine, Pittsburgh, PA USA; 3 Stanley Laboratory of Neurovirology, Department of Pediatrics, Johns Hopkins University School of Medicine, Baltimore, MD USA; 4 Department of Human Genetics, University of Pittsburgh School of Public Health, Pittsburgh, PA USA; 5 Department of Neurology, University of Pittsburgh School of Medicine, Pittsburgh, PA USA; 6 Translational Gerontology Branch, National Institute On Aging, Baltimore, MD USA; 7 Department of Epidemiology, University of Pittsburgh School of Public Health, Pittsburgh, PA USA

## Abstract

Immune processes play complex roles in Alzheimer’s disease and related dementias (ADRD), but it remains unclear whether host defenses against common microbes also contribute to ADRD, and what underlying mechanisms may account for these relationships. Using antibody and plasma proteomic data from the Monongahela-Youghiogheny Healthy Aging Team study (*n* = 1,003; mean age = 78.0 yrs; follow up 7.6 yrs), the present study detected accelerated decline across a variety of cognitive domains and altered plasma abundance of FLT1 (also known as Vascular Endothelial Growth Factor Receptor 1) among participants with elevated cytomegalovirus (CMV) titers. After analyses in the Baltimore Longitudinal Study of Aging (*n* = 323) and the UK Biobank (*n* = 956) revealed CMV titer associations with higher cross-sectional cognition and greater cognitive decline were moderated by plasma FLT1 abundance, we leveraged two-sample Mendelian Randomization to uncover causal roles for host immune responses to CMV, including limiting brain amyloidosis. Using multi-cohort proteomic signatures of CMV antibody levels and plasma FLT1, bioinformatic analyses suggested that host immune responses to CMV may impact neurologic health through alterations in lymphocytic immunoregulatory cascades, with circulating FLT1 abundance capturing the extent to which these effects may be transmitted across the blood–brain-barrier. In addition to detecting pleiotropic associations of CMV titers with neurocognitive outcomes, our findings highlight FLT1 as an important molecular moderator of these effects, and extend our understanding of the biological basis by which host immune responses to common microbes may contribute to ADRD.

**Supplementary Information:**

The online version contains supplementary material available at 10.1186/s12974-026-03800-8.

## Introduction

Systemic and central nervous system (CNS) immune processes play important roles in Alzheimer’s disease (AD) and related dementias (ADRD), but these roles are often pleiotropic and disease stage-dependent [1]. For example, biofluid and PET evidence suggests heightened immune responses can exert benefits during pre-clinical or prodromal stages of neurodegeneration, but also induce consequences with more advanced pathological burden (e.g., efficient vs impaired proteostasis) [2–5]. Despite advances in our understanding of these complex and temporally dynamic relationships, the potential contributions of microbes in ADRD, and the underlying mechanisms that may account for such contributions, require further investigation.

Hospitalized infections (e.g., sepsis) can increase ADRD risk over time, and we recently detected accelerated rates of regional brain atrophy among individuals with symptomatic viral infections [6, 7]. However, serological assays of more common, often asymptomatic infections have shown variable results in cross-sectional analyses, including evidence of lower ADRD odds and higher brain volumes among individuals with elevated antibody titers [8, 9]. Although it is unlikely that bacterial, viral, fungal, or parasitic exposures confer benefits for neurologic health, these patterns could suggest that individuals predisposed to mount a more robust immune response to common microbes (i.e., higher antibody levels due to host genetic factors and/or differences in exposures) may be more or less susceptible to neurodegeneration depending on disease stage.

Proteins in peripheral circulation may function as molecular conduits by which host immune responses to microbes contribute to variation in neurobiological processes because they can i) reflect host immunological states and change in response to inflammatory insults, ii) interact with target cells of the brain and blood–brain-barrier (BBB) through a variety of mechanisms, and iii) influence brain structure and function [10]. For example, altered plasma concentrations of inflammatory proteins following severe acute respiratory syndrome coronavirus 2 (SARS-CoV-2) infection can impact cognitive functioning and brain structure by engaging with neurovascular, immune, and endothelial cells, while changes in the broader plasma proteome following other types of microbial exposures can influence dementia risk, brain volume loss, and ADRD biomarkers [11–13]. Recent advances in high-throughput proteomic platforms have facilitated these insights by identifying reliable, scalable predictors and determinants of neurodegeneration [14]. Given their relatively high prevalence and reported associations with neurologic health, examining the plasma proteomic signatures of herpesviruses and other transmissible parasites may reveal specific molecular conduits that link host immune responses to neurodegeneration [15–17].

To determine whether host immune responses to certain microbes may contribute to ADRD, and apply plasma proteomics for illuminating the underlying mechanisms that may account for these relationships, the present study initially characterized the neurocognitive profiles of four antibody titers (CMV, HSV-1, HSV-2, and *Toxoplasma gondii*) in a discovery cohort of older adults, the Monongahela-Youghiogheny Healthy Aging Team (MYHAT) study [18]. After identifying accelerated decline across a variety of cognitive domains among MYHAT participants with elevated CMV titers, plasma proteomics from a later study visit revealed these participants would go on to exhibit altered abundance of FLT1 (also known as Vascular Endothelial Growth Factor Receptor 1; predominantly expressed in microglia and endothelial cells). Secondary analyses in two external cohorts (the Baltimore Longitudinal Study of Aging [19] [BLSA], the United Kingdom Biobank [20] [UKB]) provided evidence that CMV titers were associated with higher cognitive functioning cross-sectionally and greater cognitive decline over time, with these relationships moderated by plasma FLT1. Two-sample Mendelian Randomization (MR) analyses identified protective functions for host immune responses to CMV, including resistance to brain amyloidosis. Complementary bioinformatic analyses applied to the multi-cohort proteomic signatures of CMV titers and plasma FLT1 abundance delineated biological pathways by which host immune responses to CMV may contribute to neurologic health (e.g., natural killer cell cytotoxicity, T cell activation), as well as biological pathways by which plasma FLT1 levels may moderate these effects (e.g., chemotaxis, cell migration/motility). Given the observed effect modification and a proteomic signature enriched with BBB-specific proteins, we postulate that circulating FLT1 abundance may capture the extent to which CMV’s effects on lymphocytic immunoregulatory cascades and brain health can be transmitted across the BBB.

## Methods and materials

### Study samples (MYHAT, BLSA, GNPC, UKB)

The current investigation utilized the Monongahela-Youghiogheny Healthy Aging Team (MYHAT) study for discovery analyses, followed by the Baltimore Longitudinal Study of Aging (BLSA), the Global Neurodegeneration Proteomics Consortium (GNPC), and the United Kingdom Biobank (UKB) for external validation analyses. MYHAT is a population-based study in southwestern, Pennsylvania designed to characterize the neurocognitive profiles of older adults. The MYHAT protocols were approved by the Institutional Review Board (IRB) of the University of Pittsburgh (19020264, 19040058). Participants enrolled in MYHAT and provided blood samples between 2006–2008, which was considered their baseline visit. MYHAT participants returned for follow up visits annually, and a subset provided an additional blood sample between 2017–2020. Cognitive performance (i.e., a neuropsychological battery) was assessed at baseline and each follow up visit. BLSA is a community-based study in Baltimore, Maryland designed to assess the physical and cognitive functioning of community-dwelling older adults. The BLSA protocol was approved by the IRB of the National Institute of Environmental Health Science, National Institutes of Health (03AG0325). Due to BLSA’s continuous enrollment, participants enrolled at different times; blood samples provided between 2008–2010 were used in the current study, at which time neuroimaging and cognitive status (i.e., dementia) data were collected. The GNPC is a public–private partnership consisting of more than 20 cohorts who have contributed to a harmonized proteomic dataset derived from healthy aging, Alzheimer’s disease (AD), Parkinson’s disease, amyotrophic lateral sclerosis, and frontotemporal dementia patient samples (blood, CSF). GNPC protocols were approved by each cohort’s respective IRBs. Blood and CSF samples collected concurrently were used in the current analyses. The UKB is a population-based study in the United Kingdom designed to examine genetic, health, and lifestyle data from over half a million participants. The UKB protocol was approved by the National Health Service National Research Ethics Service (11/NW/0382). UKB participants enrolled in the study and provided blood samples between 2006–2010, which was considered their baseline visit. Cognitive status (i.e., dementia) was assessed at baseline through 09/09/2024. Antibody measurements in MYHAT used blood samples collected at baseline (i.e., 2006–2008); plasma proteomics in MYHAT used blood samples collected from a subset of individuals at a later study visit (i.e., 2017–2020). Antibody measurements and plasma proteomics in BLSA and UKB used blood samples collected at the same study visit. Informed consent was obtained from all participants and deidentified data were used for analyses. This manuscript follows Strengthening the Reporting of Observational Studies in Epidemiology guidelines [21].

### Antibodies (MYHAT, BLSA, UKB)

Antibody measurements were obtained for MYHAT [22], BLSA [8], and UKB [23], as previously described. In brief, MYHAT and BLSA samples were assayed at the Stanley Laboratory of Neurovirology (Johns Hopkins University School of Medicine) using solid-phase enzyme assays for immunoglobulin G (IgG) antibody detection. MYHAT assays employed kits from IBL America (*Toxoplasma gondii* [Toxo] and cytomegalovirus [CMV]) and Focus Diagnostics (herpes simplex virus 1 [HSV-1] and herpes simplex virus 2 [HSV-2]). BLSA assays employed kits from IBL America (CMV). As the target antigens, the assays employed purified glycoproteins for HSV-1 and HSV-2 and whole organisms for CMV and Toxo. UKB samples were assayed at the Division of Infections and Cancer Epidemiology (German Cancer Research Center) using a bead-based glutathione S-transferase (GST) capture assay for IgG antibody detection (i.e., PEPperPRINT), which incorporates glutathione-casein coated fluorescence-labelled polystyrene beads and pathogen-specific GST-X-tag fusion proteins as antigens. The UKB Infectious Disease Working Group used CMV pp28, pp52, and pp150 measurements to derive serostatus, which was validated against gold standard assays [23].

### Plasma proteomics (MYHAT, BLSA, GNPC, UKB)

Plasma proteomic data were obtained using methods previously described for MYHAT [24], BLSA [8], UKB [25], and GNPC [26]. In MYHAT, 116 targets were measured using the NULISAseq CNS Disease Panel on the Alamar Argo platform across two different plates; FLT1’s intra- and inter-plate coefficients of variation (CV) were 10.5% and 16.5%, respectively, based on a control sample duplicated across both plates. The NULISAseq CNS Disease Panel is an ultra-sensitive, high-multiplex immunoassay that measures biomarkers associated with neurodegenerative conditions and their underlying pathophysiology, including amyloid and tau pathologies (e.g., Aβ, pTau217), synaptic functioning (e.g., BDNF, SOD1), neurodegeneration (e.g., NFL, NPTX1), and inflammation (e.g., TREM2, IL6) [27]. In BLSA, 7,268 targets were measured using the v4.1 assay on the SomaScan platform; using 102 blind duplicates, FLT1’s intra-assay CV was 4.0%. In GNPC, 7,596 targets were measured using the v4.1 assay on the SomaScan platform. The SomaScan assay, developed by SomaLogic Inc (Illumina), is a high-throughput, aptamer-based proteomics platform that uses modified DNA aptamers to quantify thousands of proteins which reflect a broad range of biological processes and pathological mechanisms [28]. In UKB, 2,923 targets were measured using the Explore 3072 assay on the Olink platform; using 1,276 blind duplicates (i.e., two duplicates per plate; 628 plates for *n* = 54,219), FLT1’s intra-assay CV was 5.2%. The Olink Explore 3072 is a Proximity Extension Assay coupled with next-generation sequencing that measures thousands of proteins across diverse biological pathways [29]. Data were subjected to standardized quality control and established processing procedures in each cohort (e.g., sample exclusion based on manufacturer recommendation) [8, 24–26]. Data were analyzed on a log_2_ scale.

### CSF proteomics (GNPC)

CSF proteomic data were obtained using methods previously described for GNPC [26]. In GNPC, 7,596 targets were measured using the v4.1 assay on the SomaScan platform. Data were subjected to standardized quality control and established processing procedures in GNPC. Data were analyzed on a log_2_ scale.

### Cognitive performance (MYHAT)

A neuropsychological battery was applied in MYHAT [30]. General cognitive functioning was assessed with the Mini-Mental State Examination (MMSE). Composite scores of attention, executive function, language, memory, and visuospatial domains were also assessed, where scores from individual tasks were standardized (converted to a z score using the baseline mean and standard deviation) and averaged within each cognitive domain. Attention was assessed with digit span forward (max digit span) and Trail Making Test A (points correctly connected/time in seconds). Executive function was assessed with clock drawing (total score), Trail Making Test B (points correctly connected/time in seconds), and verbal fluency for letters P&S (average of total P & S words). Language was assessed with the IU Token test (total score), semantic verbal fluency (total score, animals), and the Boston Naming Test (sum of correct items spontaneously named or with stimulus cue); after 2/15/09, the Boston Naming Test was calculated with 59 items. Memory was assessed with Fuld Object Memory Evaluation (sum of 3 learning trials), Wechsler Memory Scale Logical Memory immediate and delayed recall, and Visual Reproduction (which was used to estimate Face Name score); after 2016, the 12-item Face Name Associative Memory Exam (sum of three sub-scores) was used for domain score calculation. Visuospatial abilities were assessed with Wechsler Adult Intelligence Scale–III Block Design.

### Dementia diagnosis (BLSA, UKB)

Dementia status was assessed in the BLSA [8] and UKB [31]. In the BLSA, participant serial clinical and neuropsychological data were reviewed at each consensus case conference if the participant had > 3 errors on the Blessed Information-Memory-Concentration test or ≥ 0.5 total combined score on the Clinical Dementia Rating scale. Dementia was based on the criteria outlined in the Diagnostic and Statistical Manual of Mental Disorders, Third Edition, and the National Institute of Neurological and Communicative Disorders and Stroke-Alzheimer’s Disease and Related Disorders Association. In the UKB, dementia was ascertained with linked hospital discharge records, primary care records, and death certificates using medical diagnostic codes for all-cause dementia (ACD) and Alzheimer’s disease (AD); the first record of diagnosis was used to define date of disease onset.

### 3 T MRI (BLSA)

T_1_-weighted magnetization-prepared rapid gradient echo scans were acquired on a 3 T Philips Achieva (repetition time [TR] = 6.8 ms, echo time [TE] = 3.2 ms, flip angle = 8°, image matrix = 256 × 256, 170 slices, pixel size = 1 × 1 mm, slice thickness = 1.2 mm, sagittal acquisition). A validated, Multi-atlas Region Segmentation Utilizing Ensembles anatomic labeling method was applied [32]. Voxel-wise maps for different brain tissue types were also calculated using the validated Regional Analysis of Volumes Examined in Normalized Space methodology; a t-value threshold of 2.0 and cluster extent of 100 voxels was used to define significant clusters [33]. Using volumes of MUSE-segmented brain regions as input features, we leveraged a semi-supervised representation learning via generative adversarial networks approach (Surreal-GAN) to calculate five R indices, which primarily reflect atrophy in subcortical (R1), medial-temporal lobe (R2), parieto-temporal lobe (R3), diffuse cortical (R4), and perisylvian (R5) regions. By distinguishing heterogenous brain volume differences between younger (< 50 years old) and older (≥ 50 years old) adults, this approach generates multiple, continuous, low-dimensional scores that reflect the co-expression level of respective brain atrophy dimensions, and accounts for simultaneous spatial and temporal disease heterogeneity within the same individual. These scores have been pretrained and validated in a diverse cohort across 11 studies (> 49,000 participants), where they predicted age-related clinical traits and disease diagnoses [34].

### Positron emission tomography (BLSA)

^11^C-Pittsburgh compound-B distribution volume ratios (DVR) of amyloid beta (Aβ) were measured using PET on a GE Advance or a Siemens High Resolution Research Tomograph scanner immediately following an injection of approximately 555 MBq of the radiotracer [35]. Mean cortical Aβ reflected the average DVR values across the cingulate, frontal, parietal (including precuneus), lateral temporal, and lateral occipital regions, excluding the pre- and post-central gyri. Mean cortical DVR values were harmonized between the two scanners by leveraging longitudinal data available on both scanners for 79 participants. Aβ status (±) was defined based on a Gaussian mixture model threshold of 1.064 mean cortical DVR [35].

### Two-sample Mendelian Randomization (MR)

For exposures, CMV titer quantitative trait loci were identified in a GWAS of plasma CMV seropositivity (*n* = 9,724; cases = 5,045; 27 QTLs at *p* < 5.0 × 10^–6^) and plasma protein quantitative trait loci were obtained from a GWAS of plasma FLT1 abundance on the SomaScan platform (*n* = 35,559; 2 QTLs at *p* < 1.8 × 10^–9^) [36, 37]. For outcomes, we used GWAS summary statistics of Alzheimer’s disease (AD; *n* = 63,926; cases = 21,982) [38], all-cause dementia (ACD; *n* = 216,771; cases = 7,284) [39], general cognitive performance (GCP; *n* = 257,841) [40], and Aβ PET (*n* = 13,409) [41]. SNPs were pruned to remove variants in linkage disequilibrium (*r*^2^ < 0.05) with the 1000 Genomes Project (European) as the reference panel. The random effects inverse variance-weighted estimate was considered for primary analyses. Sensitivity analyses were performed to assess violations of MR assumptions using the MR Egger, simple mode, weighted median, and weighted mode tests. Heterogeneity between causal estimates was tested using Cochran’s Q (MR Egger and inverse variance-weighted), and horizontal pleiotropy was tested using the MR Egger intercept. If violations of MR pleiotropy assumptions were detected, MR Egger estimates were considered for primary analyses.

### Biological characterization

A variety of analytic tools, techniques, and open-source databases were used to understand the biological implications and functional relevance of results. Tissue-specific enrichment used published findings that mapped organ-specific proteomes using human organ bulk RNA-seq data from the Genotype-Tissue Expression project (GTEx; https://gtexportal.org/home/); a cognate gene encoding a plasma protein was considered enriched if it was expressed at least four times higher in a single organ (or groups of organs) compared to any other organ [42]. Cell-specific enrichment utilized published findings that mapped cell-specific proteomes using single-cell RNA sequencing data from the Human Protein Atlas (HPA; https://www.proteinatlas.org); a cognate gene encoding a protein was considered enriched if it was expressed at least two standard deviations higher in a single cell type compared to its relative expression across all other cell types [43]. For assessing expression across cell and tissue types, we used RNA sequencing data from the HPA and GTEx. Protein interaction networks and enriched pathways were assessed using STRING (Search Tool for the Retrieval of Interacting Genes/Proteins; https://string-db.org), a database and visualization tool that integrates publicly available sources of information to provide a comprehensive understanding of protein–protein interaction (PPI) networks [44]. PPI p values derived from an explicit null model to account for the non-uniform distribution of the connectivity degrees of network proteins (i.e., a random graph with given degree sequence model) reflected the likelihood of proteins having more interactions among themselves than what would be expected for a random set of proteins of the same size drawn from the genome, suggesting if the proteins are at least partially biologically connected as a group. For enrichment analyses, strength described the size of the effect, reflecting the ratio between the number of proteins in our network that are annotated with a term and the number of proteins that would be expected to be annotated with this term in a random network of the same size. Signal was defined as a weighted harmonic mean between the observed/expected ratio and -log(False Discovery Rate, FDR); FDR tends to emphasize larger terms due to their potential for achieving lower p values, while the observed/expected ratio highlights smaller terms, which have a high foreground to background ratio but cannot achieve low FDR values due to their size. Enrichment analyses also used publicly available databases via the Enrichr platform (https://maayanlab.cloud/Enrichr/) [45]. In Enrichr, FDR adjusted p values derived from Fisher’s exact tests quantified the probability of overlap between cognate genes encoding proteins and molecules known to exist within a specific term due to random chance. The combined scores reflected the product of p values (log transformed) from Fisher’s exact tests multiplied by the z-scores of the deviations from each gene’s expected rank, which were derived from randomly imputed gene sets for each term. Supplementary information on individual SNPs was obtained with the OpenTargets (https://www.opentargets.org) and GTEx platforms.

### Covariates

Age (years), sex (male/female), race (white/non-white), and education were defined based on self-report in MYHAT, BLSA, and UKB. Education was quantified continuously (years) in MYHAT and BLSA, and as levels in UKB (A levels/AS levels or equivalent, college or university degree/CSEs, O Levels/NVQ, HND, HNC, or equivalent/none of the above/prefer not to answer/other professional qualifications). In MYHAT and BLSA, *APOE4* carrier status (0 ε4 alleles/≥ 1 ε4 alleles/missing) was defined via PCR with restriction isotyping using the Type IIP enzyme Hhai or a Taqman assays. In the UKB, *APOE4* carrier status (0 ε4 alleles/≥ 1 ε4 alleles/1/3 or 2/4 alleles) was defined from variants rs7412 and rs429358 assessed on the Affymetrix Axiom/BiLEVE microarray platform.

### Statistical analyses

A hierarchical gating approach was employed for statistical analyses, whereby antibodies and proteins of interest for downstream analyses were first identified with discovery analyses in MYHAT using both nominally significant and FDR-corrected p values of 0.05; here, FDR corrections were applied per antibody titer. This was followed by the utilization of external cohorts (BLSA, UKB) and orthogonal strategies to reduce the possibility of Type I error, where nominally significant p values of 0.05 were applied (i.e., FDR correction was not applied for external cohorts). Consistent with prior procedures [8], continuous titer measurements and titer tertiles were examined as predictors in MYHAT and BLSA; this analytic approach was favored over examining serostatus, as older adults can have high rates of seropositivity and meaningful cut points among these individuals can be unreliable [46]. Consistent with prior procedures [47], we prioritized examining serostatus as a predictor in UKB, which was previously defined and validated by the UKB Infectious Disease Working Group using CMV pp28, pp52, and pp150 IgG measurements [23]; additionally, continuous and tertile pp28, pp52, and pp150 measurements were examined as predictors. Covariates were selected based on theory-driven rationale as well as data availability across cohorts. Participants with missing predictor, outcome, and/or covariate data were not included in analyses. Analyses were conducted separately in each cohort for each outcome of interest due to the inherently heterogenous study designs, data structures, and outcome distributions between cohorts.

In MYHAT, multiple linear regression models adjusted for baseline age, sex, race, education, and *APOE4* were used to examine associations of titers with cross-sectional differences in cognitive performance. Linear mixed effects models adjusted for the aforementioned covariates and their interactions with time were used to examine associations of titers with longitudinal rates of change in cognitive performance. Given the limited sample size, minimally adjusted multiple linear regression models adjusted for baseline age, sex, and time difference between blood draws (i.e., time between blood draw used for antibody measurement in 2006–2008 and blood draw used for proteomic measurement in 2017–2020) were used to examine associations of baseline titers and future differences in plasma proteomic abundance. In BLSA, the same covariates were used as in MYHAT. Here, multiple linear regression models adjusted for baseline age and sex were used to examine associations of titers with differences in plasma proteomic abundance. Multiple linear regression models adjusted for baseline age, sex, race, education, and *APOE4* were used to examine associations of titers with brain structure; voxel-based analyses additionally adjusted for intracranial volume. Logistic regression models adjusted for baseline age, sex, race, education, and *APOE4* were used to examine associations of titers with odds of dementia. In UKB, the same covariates were used as in MYHAT. Here, multiple linear regression models adjusted for baseline age and sex were used to examine associations of titers with differences in plasma proteomic abundance. Cox proportional hazards regression models adjusted for baseline age, sex, race, education, and *APOE4* were used to examine associations of titers with risk of dementia (i.e., ACD, AD). In BLSA and UKB, separate statistical models were used to investigate associations of CMV titers, FLT1, and their interactions (i.e., CMV*FLT1) with cognition. Interaction analyses were conducted to determine whether CMV titer associations with cognition were moderated by plasma FLT1 abundance. Multiple linear regression models adjusted for baseline age and sex were used to examine associations of CMV titers and FLT1 with differences in plasma proteomic abundance (i.e., proteomic signatures of CMV titers and FLT1 in BLSA and UKB); to identify the most robust associations across cohorts, cohort specific effects (i.e., effect size [beta] estimates from BLSA and effect size [beta] estimates from the UKB) were aggregated in fixed- and random-effects inverse variance-weighted meta-analyses. Spearman correlations were used to examine cross-biofluid correlations in GNPC. FDR correction used the Benjamini–Hochberg procedure. Model quality and goodness of fit were assessed using the performance package (0.15.2). Proportional hazards assumptions were assessed with Kaplan Meier curves and Schoenfeld residuals. Analyses were performed in R (4.2.2). Graphs were generated in R, STRING, and BioRender.

## Results

### CMV titers predict faster cognitive decline

**Fig. 1:**
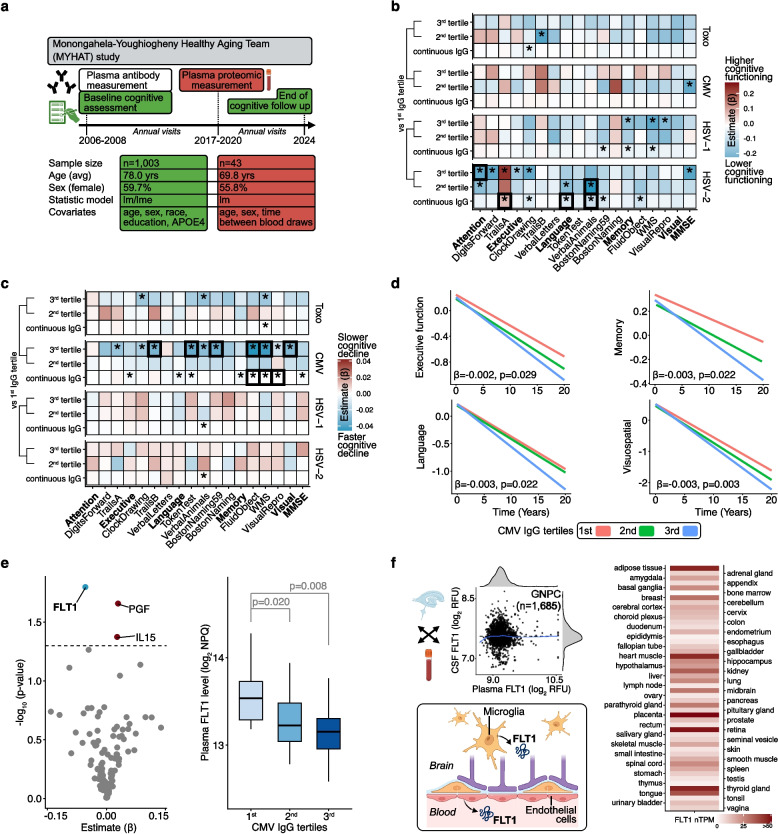
Discovery analyses. **a** Overview of MYHAT analyses. **b** Cross-sectional cognitive performance associations with continuous and tertile antibody titers. Composite scores of five cognitive domains and one measure of general cognitive functioning are bolded; individual task components used to derive composite scores are listed adjacent to their respective domains. Results derived from linear regression models adjusting for age, sex, race, education, and *APOE4*. *indicates *p* < 0.05. Bolded tiles indicate FDR < 0.05. **c** Longitudinal cognitive performance associations with continuous and tertile antibody titers. Composite scores of five cognitive domains and one measure of general cognitive functioning are bolded; individual task components used to derive composite scores are listed adjacent to their respective domains. Results derived from linear mixed effects models adjusting for age, sex, race, education, *APOE4*, and their interactions with time. *indicates *p* < 0.05. Bolded tiles indicate FDR < 0.05. **d**. Longitudinal cognitive performance associations with tertile CMV antibody titers. Results derived from linear mixed effects models adjusting for age, sex, race, education, *APOE4*, and their interactions with time. βs and corresponding p values represent differences in estimated annual cognitive performance changes associated with continuous CMV antibody titers. **e** Plasma protein associations with continuous CMV antibody titers; plasma FLT1 associations with tertile CMV antibody titers. In the volcano plot, proteins above the dashed horizontal line were statistically significant (*p* < 0.05) and labeled. Results derived from linear regression models adjusting for age, sex, and time difference between blood draws (i.e., time between blood draw used for antibody measurement in 2006–2008 and blood draw used for proteomic measurement in 2017–2020). **f** FLT1 cross-biofluid (CSF, plasma) correlation; FLT1 tissue/cell specific expression**.** CSF and plasma FLT1 data were obtained from the Global Neurodegeneration Proteomics Consortium (GNPC). Single cell and bulk RNA sequencing (normalized transcripts per million; nTPM) were obtained from the Human Protein Atlas

To determine whether host immune responses to certain microbes may also be associated with cognitive functioning in a population-based cohort of older adults, we examined whether HSV-1, HSV-2, CMV, and Toxo IgG titers measured with solid-phase enzyme assays were related to cross-sectional differences and longitudinal rates of change in cognitive performance among Monongahela-Youghiogheny Healthy Aging Team (MYHAT) study participants (*n* = 1,003; mean age = 78.0 yrs. [SD = 7.5]; 59.7% female; 4.4% non-white; Table 1; sTable 1; sFigure 1; Fig. 1a). To detect broad, consistent patterns with cognitive performance, we examined associations with the complete neuropsychological battery, which included a measure of general cognitive functioning (MMSE) as well as composite scores of five cognitive domains (i.e., attention, executive function, language, memory, visuospatial domains) and their individual task components. The average follow-up time for longitudinal analyses was 7.6 years (median: 7.0, IQR = 3.0, 12.0) with an average of 8.4 (SD = 4.7) cognitive assessments per participant (range: 2–17). Distributions of CMV and HSV-1 titer data showed minor positive skews, whereas Toxo and HSV-2 titer data displayed strong positive skews (sFigure 2).

**Table 1:** Sample characteristics

	MYHAT (*n* = 1,003)	GNPC (*n* = 1,685)	BLSA (*n* = 323)	UKB (*n* = 956)
Age (yrs)	78.0 (7.5)	71.2 (9.4)	77.6 (10.1)	57.1 (8.3)
Women	599 (59.7%)	985 (58.5%)	141 (43.7%)	536 (56.1%)
Race (white)	959 (95.6%)	100 (5.9%)	253 (78.3%)	887 (92.8%)
Race (non-white)	44 (4.4%)	14 (0.8%)	65 (20.1%)	51 (5.3%)
Black or African American	39 (3.9%)	1 (0.1%)	59 (18.3%)	0 (0.0%)
American Indian or Alaska Native	0 (0.0%)	2 (0.1%)	1 (0.3%)	0 (0.0%)
Asian	2 (0.2%)	11 (0.7%)	4 (1.2%)	19 (2.0%)
Native Hawaiian or Other Pacific Islander	0 (0.0%)	0 (0.0%)	1 (0.3%)	0 (0.0%)
More than one race	3 (0.3%)	0 (0.0%)	0 (0.0%)	8 (0.8%)
African	0 (0.0%)	0 (0.0%)	0 (0.0%)	14 (1.5%)
Caribbean	0 (0.0%)	0 (0.0%)	0 (0.0%)	10 (1.1%)
Race (unreported)	0 (0.0%)	1,571 (93.2%)	5 (0.6%)	18 (1.9%)
Education (yrs)	12.9 (2.5)	9.2 (5.3)	17.1 (2.3)	14.4 (1.8)
*APOE*ε4 alleles				
0	791 (78.9%)	891 (52.9%)	218 (67.5%)	719 (75.2%)
≥ 1	205 (20.4%)	451 (26.8%)	96 (29.7%)	208 (21.8%)

Values are displayed as means (standard deviation) and frequencies (percentages). Characteristics reflect the titer-cognition MYHAT sample, proteomic GNPC sample, the titer-cognition BLSA sample, and the titer-proteomic-cognition UKB sample. Education years in UKB were estimated based on education level (A levels/AS levels or equivalent, college or university degree/CSEs, O Levels/NVQ, HND, HNC)

In linear regression models adjusted for age, sex, race, education, and *APOE4*, HSV-2 titers were most strongly and consistently associated with cross-sectional cognitive performance, including lower scores across multiple cognitive domains and/or domain-specific cognitive tasks (Fig. 1b; sTable 2). These results align with a prior MYHAT investigation, which found lower baseline performance across each cognitive domain among HSV-2 seropositive cases [22]. Interestingly, HSV-2 titers were also linked to elevated performance on a measure of attention (i.e., Trails A). While we found lower performance on certain cognitive domains and/or tasks among participants with higher Toxo, HSV-1, and CMV titers, these associations were not statistically significant following FDR correction for multiple comparisons, whereas 30% (5/15) of HSV-2 titer associations were retained (sTable 2).

In linear mixed effects models adjusted for the aforementioned covariates and their interactions with time, CMV titers were most strongly associated with faster rates of cognitive decline across multiple domains and/or domain-specific tasks (Fig. 1c, d; sTable 3). By detecting CMV titer-related declines on measures of attention, executive function, language, and general cognitive functioning, these results add to a prior MYHAT investigation, which found faster decreases in memory and visuospatial abilities among CMV seropositive cases over a shorter, 5-year follow up period [22]. Following FDR correction for multiple comparisons, longitudinal relationships Toxo, HSV-1, and CMV titers were not statistically significant, whereas 50% (9/18) of CMV titer associations were retained (sTable 3).

CMV is a highly prevalent (50–90% seroprevalence) pantropic beta herpesvirus transmitted via biofluids (e.g., saliva) that establishes life-long latency with potential for reactivation following initial exposure, which is typically asymptomatic and often occurs during childhood [48]. Given the consistent pattern of results and persistence of longitudinal associations after correction for multiple comparisons, CMV titers were prioritized for subsequent investigation.

### CMV titers are linked to plasma FLT1

To identify potential molecular conduits by which host immune responses to CMV may be related to variation in cognitive functioning in MYHAT, we leveraged plasma proteomic data from the NULISAseq CNS disease panel (116 protein targets) among a subset of MYHAT participants (*n* = 43; mean age = 69.8 yrs. [SD = 4.4]; 55.8% female; 9.3% non-white; sTable 1; Fig. 1a) who provided an additional blood sample approximately 11.0 years (mean: 11.0, SD = 0.8, median: 10.8, IQR = 10.4, 11.5) after study enrollment (i.e., when antibody titers were measured). Using linear regression models adjusted for age, sex, and time difference between blood draws (i.e., time between blood draw used for antibody measurement in 2006–2008 and blood draw used for proteomic measurement in 2017–2020), we found baseline CMV titers were associated with future differences in plasma proteomic abundance of three proteins, namely higher IL15 (Interleukin-15; β = 0.027, *p* = 0.042), higher PGF (Placental Growth Factor; β = 0.030, *p* = 0.022), and lower FLT1 (β = −0.054, *p* = 0.016; sTable 4; Fig. 1e). Unlike IL15 and PGF, levels of FLT1 were associated with both continuous and tertile CMV titers (sTable 4; Fig. 1e). FLT1’s associations with baseline CMV titers did not survive FDR correction for multiple comparisons; however, because it demonstrated the strongest effect size and was the only protein to show consistent associations with both continuous and tertile CMV titers, this protein was prioritized for subsequent post hoc analyses.

### Characterization of FLT1

FLT1 (also known as Vascular Endothelial Growth Factor Receptor 1 or Fms Related Receptor Tyrosine Kinase 1) is a tyrosine-protein kinase that functions as a cell-surface receptor for vascular endothelial growth factor (VEGF) family members and is primarily known for modulating angiogenesis as well as inflammatory pathways [49]. Although FLT1’s expression is not organ-specific, it maintains elevated expression in thyroid, placental, and adipose tissues (sTable 5; sFigure 3; Fig. 1f). Relative to other cell types, FLT1 exhibits robust expression in microglia and endothelial cells, suggesting its abundance in peripheral circulation could reflect neuroinflammatory processes and BBB integrity (sTable 5; sFigure 3; Fig. 1f). However, we found no cross biofluid association (*rho* = 0.00, *p* = 0.99; Fig. 1f) between plasma and CSF FLT1 levels measured with SomaScan from concurrently collected plasma and CSF samples in the Global Neurodegeneration Proteomics Consortium (GNPC; *n* = 1,685; mean age = 71.2 yrs. [SD = 9.4]; 58.5% female; 0.8% non-white; Table 1; sTable 1; sFigure 4).

### FLT1 moderates the relationship of CMV titers with odds of dementia

**Fig. 2:**
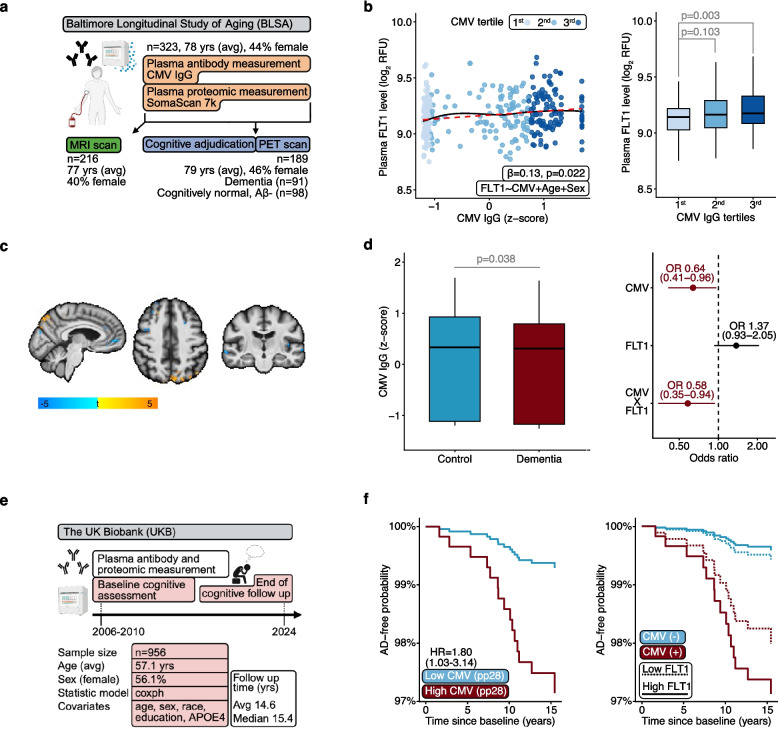
External validation of CMV and FLT1. **a** Overview of BLSA analyses. **b** Plasma FLT1 associations with continuous and tertile CMV antibody titers. Results derived from linear regression models adjusting for age and sex. In the scatterplot, lines of best fit (red, dashed) and locally weighted scatterplot smoothing (LOWESS; black, solid) are displayed, with the β and corresponding *p* value representing differences in plasma FLT1 associated with continuous CMV antibody titers. **c** Representative images show associations of CMV titers with voxel-wise differences in brain volumes. A threshold of 50 voxels with a t-value of 2.0 was used to define significant clusters. Results derived from linear regression models adjusting for age, sex, race, education, APOE4, and intracranial volume. **d** Cross-sectional cognitive status associations with continuous CMV antibody titers and plasma FLT1. Results derived from logistic regression models adjusting for age, sex, race, education, and *APOE4*. Separate statistical models were used to derive associations with CMV titers, FLT1, and their interactions (i.e., CMV*FLT1). **e** Overview of UKB analyses. **f** 15-year Alzheimer’s disease (AD) event-free probabilities grouped according to CMV antibody titer (pp28 IgG) groups (low, high; median split); event free probabilities according to plasma FLT1 levels and CMV serostatus. The hazard ratio (HR) in the Kaplan–Meier plot represents differences in AD risk associated with continuous CMV antibody titers (pp28 IgG). Results derived from Cox proportional hazards regression models adjusted for age, sex, race, education, and *APOE4*

Having identified FLT1 as a protein in peripheral circulation that may influence the relationship between host immune responses to CMV and cognitive functioning, we then asked whether CMV titers and their interaction with FLT1 abundance may be related to differences in brain structure and odds of dementia in a community-based cohort of older adults, the Baltimore Longitudinal Study of Aging (BLSA; sFigure 5; Fig. 2a). BLSA participants had CMV IgG titers measured with solid-phase enzyme assays (which showed a platykurtic distribution with a minor positive skew; sFigure 6), plasma proteomic data available from the SomaScan v4.1 assay (7,268 protein targets), and structural neuroimaging data from 3 T MRI. Clinically adjudicated cognitive status and machine learning-derived patterns of age-related brain volume loss (subcortical, medial-temporal, parieto-temporal, diffuse cortical, perisylvian) were examined as outcomes [34]. Using previously computed results from an external cohort (*n* = 44) which leveraged a single plasma sample from each participant to obtain proteomic measurements on both the NULISA (which was used to measure proteins in MYHAT) and SomaScan (which was used to measure proteins in BLSA) platforms, we noted that the correlation of FLT1 quantification across platforms is modest (*rho* = 0.34, *p* = 0.025) [50].

Using all BLSA participants with available titer and proteomic measurements (*n* = 323; mean age = 77.6 yrs. [SD = 10.1]; 43.7% female; 20.1% non-white; Table 1; sTable 1) and linear regression models adjusted for age and sex (i.e., the same covariates used in MYHAT discovery proteomic analyses), we found higher CMV titers were accompanied by greater FLT1 abundance (β = 0.129, *p* = 0.022; sTable 6; Fig. 2b). Given that CMV titers were positively associated with concurrently measured FLT1 in BLSA (i.e., BLSA titer data and SomaScan proteomic data were derived from the sample blood sample) but negatively associated with future FLT1 levels in MYHAT (i.e., MYHAT proteomic data on the NULISA platform were derived from blood draws collected approximately 11.0 years [on average] after CMV titers were measured), we conducted additional analyses to identify potential reasons for this discordance. Although a look up of the FLT1 correlation across NULISA and SomaScan assays revealed modest associations (*n* = 44; *rho* = 0.34), we examined other possible contributors as well [50]. We asked whether a subset of inflammation/vascular proteins measured on the NULISA platform (i.e., the assay used for MYHAT proteomics) and the same proteins measured on the SomaScan platform (i.e., the assay used for BLSA proteomics) were related to both FLT1 and CMV titers. We hypothesized that if FLT1 and/or CMV titers displayed discordant relationships with these proteins across MYHAT and BLSA, then such discordance might partially account for inconsistent FLT1-CMV titer relationships across cohorts. To ensure we reliably captured indicators of similar biological process, we focused on proteins with high correlations across platforms (i.e., *rho* > 0.50), as reported previously (*n* = 44; sTable 7). BLSA FLT1 abundance (and to a lesser extent, CMV titers) tended to show significant inverse associations with concurrently measured SomaScan inflammation markers, whereas MYHAT FLT1 abundance and titer levels were not strongly associated with concurrently measured NULISA inflammation or vascular markers (sFigure 7). Additional analyses revealed demographic factors and the timing of blood draws did not modify results (sTable 7). Together, these results suggest that different platforms for BLSA and MYHAT proteomic data and different inflammatory profiles between BLSA and MYHAT participants (but not other differences like demographics or timing of blood draws) may have contributed to discordant FLT1-CMV associations across cohorts.

Leveraging a subset of BLSA participants with MRI data (*n* = 216; mean age = 76.6 yrs. [SD = 9.2]; 40.3% female; 23.6% non-white; sTable 1) along with linear regression models adjusted for age, sex, race, education, and *APOE4* (i.e., the same covariates used in MYHAT discovery cognition analyses), we found higher CMV titers were also accompanied by lower diffuse cortical atrophy (β = −0.143, *p* = 0.020; sFigure 8), but this relationship was not moderated by plasma FLT1 (FLT1*CMV; β = −0.303, *p* = 0.318; sTable 8). Secondary, voxel-wise analyses showed CMV titer associations with preserved volumes were most evident in posterior cortical regions (e.g., cuneus), although greater atrophy in certain temporal regions was also detected (e.g., superior temporal gyrus; Fig. 2c; sTable 8).

Using BLSA participants who were cognitively adjudicated and had Aβ PET (dementia = 91; cognitively normal, Aβ-negative = 98; mean age = 78.7 yrs. [SD = 9.6]; 45.5% female; 22.9% non-white; sTable 1) along with logistic regression models adjusted for the aforementioned covariates, we found 36% lower odds of dementia associated with 1SD higher CMV titer (odds ratio [OR] = 0.64, 95% confidence interval [CI]: 0.41, 0.96; sTable 9; Fig. 2d). Although FLT1 itself was not associated with cognitive status (OR = 1.37, 95% CI: 0.93, 2.05), FLT1 moderated the association of CMV with dementia (FLT1*CMV; OR = 0.58, 95% CI: 0.35, 0.94; sTable 9; Fig. 2d). These results align with a prior BLSA investigation, which found lower odds of dementia associated with higher HCoV-OC43 IgG and CMV immunoglobulin M (IgM) titers [8]. Insufficient numbers of incident cognitive impairment cases in the current dataset prevented the examination of associations with dementia risk. While these findings provide evidence that CMV titers can be associated with plasma FLT1 levels across multiple cohorts (MYHAT, BLSA), they also suggest that host immune responses to CMV may be related higher cognitive functioning cross-sectionally (BLSA) but greater cognitive decline over time (MYHAT), with this relationship potentially moderated by plasma FLT1 abundance (i.e., FLT1*CMV x lower dementia odds in BLSA).

### FLT1 moderates the relationship of CMV titers with 15-year Alzheimer’s disease (AD) risk

To evaluate the relationship of CMV titers with etiology-specific dementia risk in a population-based cohort from outside the United States and determine whether plasma levels of FLT1 modify this association, we leveraged data from a subset of UK Biobank (UKB) participants (*n* = 956; mean age = 57.1 yrs. [SD = 8.3]; 56.1% female; 5.3% non-white) who provided blood samples at enrollment that were used to measure CMV IgG titers on a bead-based glutathione S-transferase capture assay and plasma proteomics on the Olink Explore assay (2,923 protein targets; Table 1; sTable 1; sFigure 9, 10; Fig. 2e). All-cause dementia (ACD) and Alzheimer’s disease (AD) were examined as outcomes. The average follow-up time was 14.6 years (median: 15.4, IQR = 14.6, 16.1). The correlation of FLT1 quantification in plasma across the NULISA (which was used to measure proteins in MYHAT) and Olink (which was used to measure proteins in UKB) platforms is not available; however, the correlation of FLT1 across these platforms in CSF is moderate, as previously reported (*n* = 44; *rho* = 0.54, *p* < 0.001) [50].

In Cox proportional hazards regression models adjusted for age, sex, race, education, and *APOE4* (i.e., the same covariates used in MYHAT discovery cognition analyses), CMV serostatus and IgG measurements of pp52 and pp150 were not related to ACD or AD risk, but we detected 80% increased risk of AD associated with 1SD higher pp28 IgG (HR = 1.80, 95% CI: 1.03, 3.14; sTable 10; sFigure 11; Fig. 2f). We also found an interaction of FLT1 and CMV titers, such that seropositive participants who had higher FLT1 abundance exhibited a multi-fold increased risk of AD over the 15 year follow up period (FLT1*CMV; HR = 5.10, 95% CI: 3.78, 6.87; sTable 10; Fig. 2f). A similar but statistically insignificant pattern was found for ACD (FLT1*CMV; HR = 2.06, 95% CI: 0.89, 4.74; sTable 10). FLT1 was not associated with ACD or AD risk (sTable 9). Insufficient numbers of prevalent cognitive impairment cases precluded cross-sectional analyses in the current dataset, but a prior study using the larger set of UKB participants with availably serology (but not proteomic) data (*n* = 9,431) found a non-significant association of CMV seropositivity with lower odds of prevalent dementia (OR = 0.89, CI: 0.57, 1.37) [47]. Although FLT1 is reportedly elevated among UKB participants with viral infections, we did not observe a relationship between CMV titers and plasma FLT1 levels in our linear regression analyses adjusted for age and sex (i.e., the same covariates used in MYHAT discovery proteomic analyses; sTable 11, 12) [51].

In conjunction with preceding MYHAT and BLSA results, these UKB results suggest host immune responses to CMV may be associated with higher cognitive functioning cross-sectionally (BLSA) but greater cognitive decline over time (MYHAT, UKB). Plasma FLT1 abundance appears to moderate the associations of CMV titers with prevalent and incident dementia.

### Causal roles of host immune responses to CMV

**Fig. 3:**
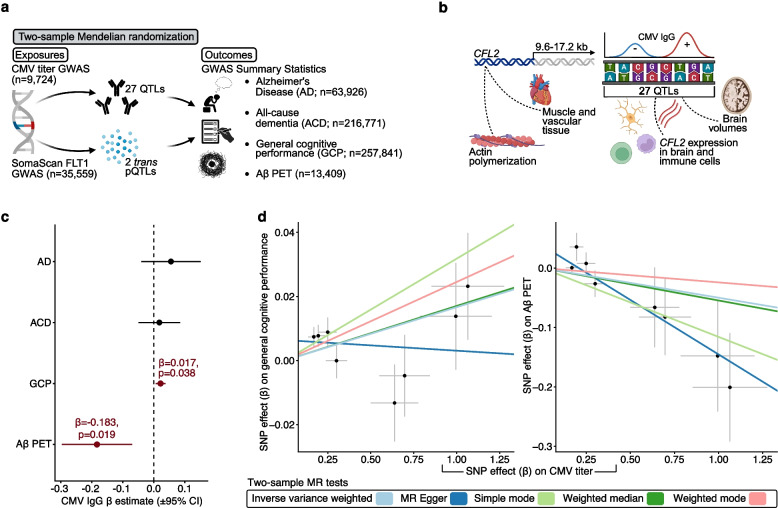
Mendelian Randomization (MR) analyses. **a** Overview of MR analyses. **b** CMV titer quantitative trait loci (QTLs) characterization and supplementary information. **c** Associations of genetically determined CMV titers with genetically determined Alzheimer’s disease (AD), all-cause dementia (ACD), general cognitive performance (GCP), and Aβ PET. CMV titer quantitative trait loci were identified in a GWAS of plasma CMV seropositivity (*n* = 9,724; cases = 5,045). The random effects inverse variance-weighted estimate was considered for primary analyses; if violations of MR assumptions were detected, MR Egger estimates were considered for primary analyses. **c** Individual SNP associations of genetically determined CMV titers with genetically determined general cognitive performance and cortical Aβ PET levels. GWAS summary statistics of general cognitive performance (*n* = 257,841) and Aβ PET (*n* = 13,409) were used to assess the outcomes

Before identifying biological pathways that may account for these observed associations, we sought to establish causal evidence for plasma FLT1 and host immune responses to CMV (Fig. 3a). To this end, we employed two-sample Mendelian Randomization (MR) after combining a GWAS of CMV seropositivity [36] and a GWAS of SomaScan plasma FLT1 abundance [37] with GWAS summary statistics of AD [38], ACD [39], general cognitive performance (GCP) [40], and Aβ PET [41] (Fig. 3a). CMV titer quantitative trait loci (QTLs; *n* = 27; *p* < 5.0 × 10^–6^) were concentrated 9.58–17.18 kb downstream of the *CFL2* gene encoding the cofilin 2 protein, which regulates actin filament dynamics and is predominantly expressed in muscle and vascular tissue; along with altered *CFL2* expression in multiple brain regions (e.g., frontal cortex, putamen etc.,) and immune cell types (e.g., T cells, monocytes, etc.,), these SNPs have been associated with higher brain volumes in prior GWAS (Fig. 3b; sTable 13, 14). Plasma FLT1 quantitative trait loci (*n* = 2; *p* < 1.8 × 10^–9^) were in *trans* to *FLT1*; while one of these SNPs is downstream from a long non-coding RNA (rs6921438), the other (rs10922098) is located in the intronic region of the *CFH* gene encoding the complement factor H protein and has been associated with altered *CFH* expression in the dorsolateral prefrontal cortex as well as in macrophages (sTable 13, 14). FLT1 results should be interpreted with caution given its *trans* genetic instruments, which suggest the limited differences in FLT1 abundance attributed to underlying genetic variation are likely mediated through indirect mechanisms (e.g., unknown transcription factors near pQTLs which influence expression of FLT1).

We found evidence supporting a causal relationship of higher CMV titers with higher GCP (β = 0.017, *p* = 0.038) and lower cortical Aβ PET levels (β = −0.183, *p* = 0.019; sTable 15, 16; sTable 12, 13; Fig. 3c, d). FLT1 was not linked to neurocognitive outcomes. These findings support a causal, protective role for host immune responses to CMV, including for limiting brain amyloidosis.

### CMV titer and plasma FLT1 proteo-biological signatures

**Fig. 4:**
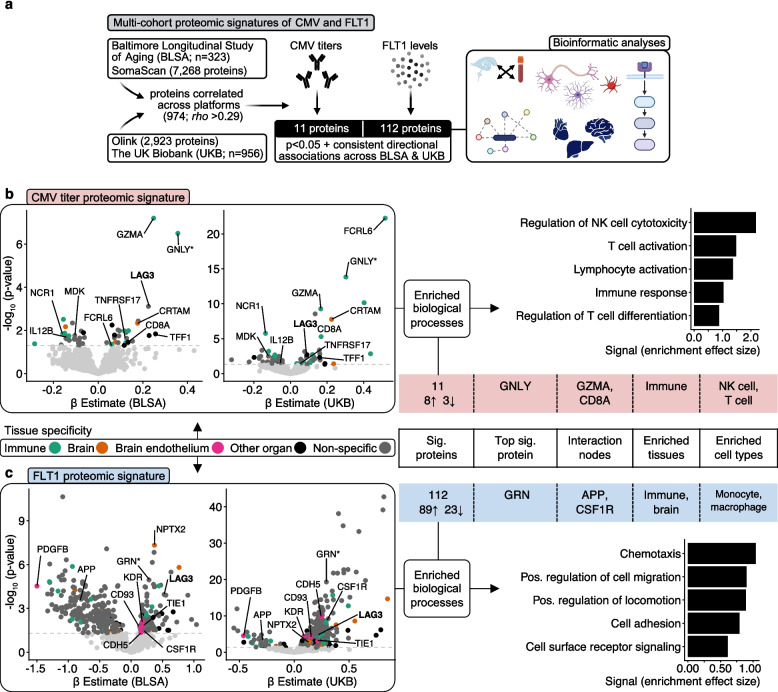
CMV titer and plasma FLT1 proteo-biological signatures. **a** Overview of proteomic and bioinformatic analyses. **b** CMV titer proteomic signatures and bioinformatic results. In the volcano plots, proteins above the dashed horizontal line were statistically significant (*p* < 0.05); plasma proteins that maintained consistent directional associations with CMV titers across BLSA and UKB are labeled. *indicates the protein which illustrated the strongest statistical association in meta-analyzed results. Proteomic results derived from linear regression models adjusting for age and sex. The top significant protein was identified using cohort specific effects in random-effects inverse variance-weighted meta-analysis. Cell- and tissue-specific enrichment used expression data from the Genotype-Tissue Expression project and the Human Protein Atlas. Protein interaction networks were assessed using STRING (Search Tool for the Retrieval of Interacting Genes/Proteins). Enrichment analyses used publicly available databases via STRING and Enrichr platforms. **c** Plasma FLT1 proteomic signatures and bioinformatic results. In the volcano plots, proteins above the dashed horizontal line were statistically significant (*p* < 0.05); the top plasma proteins that maintained consistent directional associations with plasma FLT1 across BLSA and UKB are labeled. *indicates the protein which illustrated the strongest statistical association in meta-analyzed results. Proteomic results derived from linear regression models adjusting for age and sex. The top significant protein was identified using cohort specific effects in random-effects inverse variance-weighted meta-analysis. Cell- and tissue-specific enrichment used expression data from the Genotype-Tissue Expression project and the Human Protein Atlas. Protein interaction networks were assessed using STRING (Search Tool for the Retrieval of Interacting Genes/Proteins). Enrichment analyses used publicly available databases via STRING and Enrichr platforms

To identify biological pathways by which host immune responses to CMV may be contributing to neurologic health, as well as biological pathways by which plasma FLT1 levels may be moderating these effects, we applied complementary bioinformatic analyses to their multi-cohort proteomic signatures (Fig. 4a). Using linear regression models adjusted for age and sex, plasma proteins that maintained consistent directional associations with CMV titers (i.e., 1 st vs 3rd tertile in BLSA, seronegative vs seropositive in UKB) or FLT1 abundance at a nominally significant *p* value (*p* < 0.05) across BLSA (*n* = 323) and UKB (*n* = 956) were used in downstream bioinformatic analyses. To ensure results were as reliable as possible across cohorts, we restricted proteome-wide analyses to measurements exceeding plasma FLT1’s reported correlation across the SomaScan (BLSA) and Olink (UKB) platforms (*rho* = 0.29, *p* < 0.001), which included 974 unique protein targets [52] (sTable 17).

We detected 11 plasma proteins that showed consistent directional associations with CMV titers across BLSA and UKB (3 negative and 8 positive associations; sTable 18; Fig. 4b). Using plasma and CSF SomaScan data from GNPC (*n* = 1,685), their average cross-biofluid correlation was modest (average *rho* = 0.27; range: 0.02–0.60; sTable 19). In this proteomic signature of CMV titers, we found enrichment of immune tissue-, natural killer (NK) cell-, and T cell-specific proteins, including granulysin (*GNLY*; a cytotoxic antimicrobial peptide), which exhibited the strongest statistical association in meta-analyzed results (sTable 18–20; sFigure 14). Protein interaction analyses revealed a robust pattern of co-expression, including focal nodes of Granzyme A (*GZMA*; a protease that facilitates lymphocyte-mediated apoptosis of virally infected cells) and Cluster of Differentiation 8 (*CD8A*; a T cell facilitator of antigen recognition; sTable 21; sFigure 15). Pathway analyses indicated a consistent induction of lymphocytic immunoregulatory cascades and lymphocyte phenotypic remodeling, including NK cell mediated cytotoxicity, CD8 + T cell activation, and Major Histocompatibility Complex (MHC) signaling (sTable 22; sFigure 16; Fig. 4b).

We also identified 112 plasma proteins that showed consistent directional associations with plasma FLT1 abundance across cohorts (23 negative and 89 positive associations; sTable 23; Fig. 4c). Using GNPC, the average cross-biofluid correlation of these proteins was limited (average *rho* = 0.15; range: 0.00–0.70; sTable 24). FLT1’s proteomic signature was enriched with immune tissue-, monocyte/macrophage-, and CNS cell- (i.e., oligodendrocyte, neuron) specific proteins, including Progranulin (*GRN*; contributes to autosomal dominant frontotemporal dementia), which illustrated the strongest statistical association in meta-analyzed results (sTable 23–25; sFigure 17, 18). Robust associations with previously documented biomarkers of brain endothelial cells (PDGFB, KDR, TIE1, CDH5, CD93) were also detected; along with its predominant expression in microglia and endothelial cells (Fig. 1f), these findings further suggest that FLT1 abundance in plasma may reflect BBB integrity [43]. Protein interaction analyses revealed an extensive interconnected network in FLT1’s proteomic signature, including key nodes of Amyloid Precursor Protein (*APP*; necessary for Aβ synthesis) and Colony Stimulating Factor 1 receptor (*CSF1R*; critical for microglial functioning; sTable 26; sFigure 19). While pathway analyses revealed enrichment of several CNS-specific processes (e.g., axon guidance, synapse structure, etc.,), we also detected consistent enrichment of mechanisms underlying cellular transmigration, with many immunological proteins also playing roles in chemotaxis and chemoattraction, as well as cell migration and motility (sTable 27; sFigure 20; Fig. 4c). One protein, Lymphocyte Activation Gene 3 (*LAG3*), maintained consistently greater abundance among participants with elevated CMV titers and FLT1 levels (Fig. 4b, c); with enhanced expression in the choroid plexus, NK cells, and T cells, this transmembrane protein’s canonical functions include the regulation of T cell activation and cell adhesion.

**Fig. 5:**
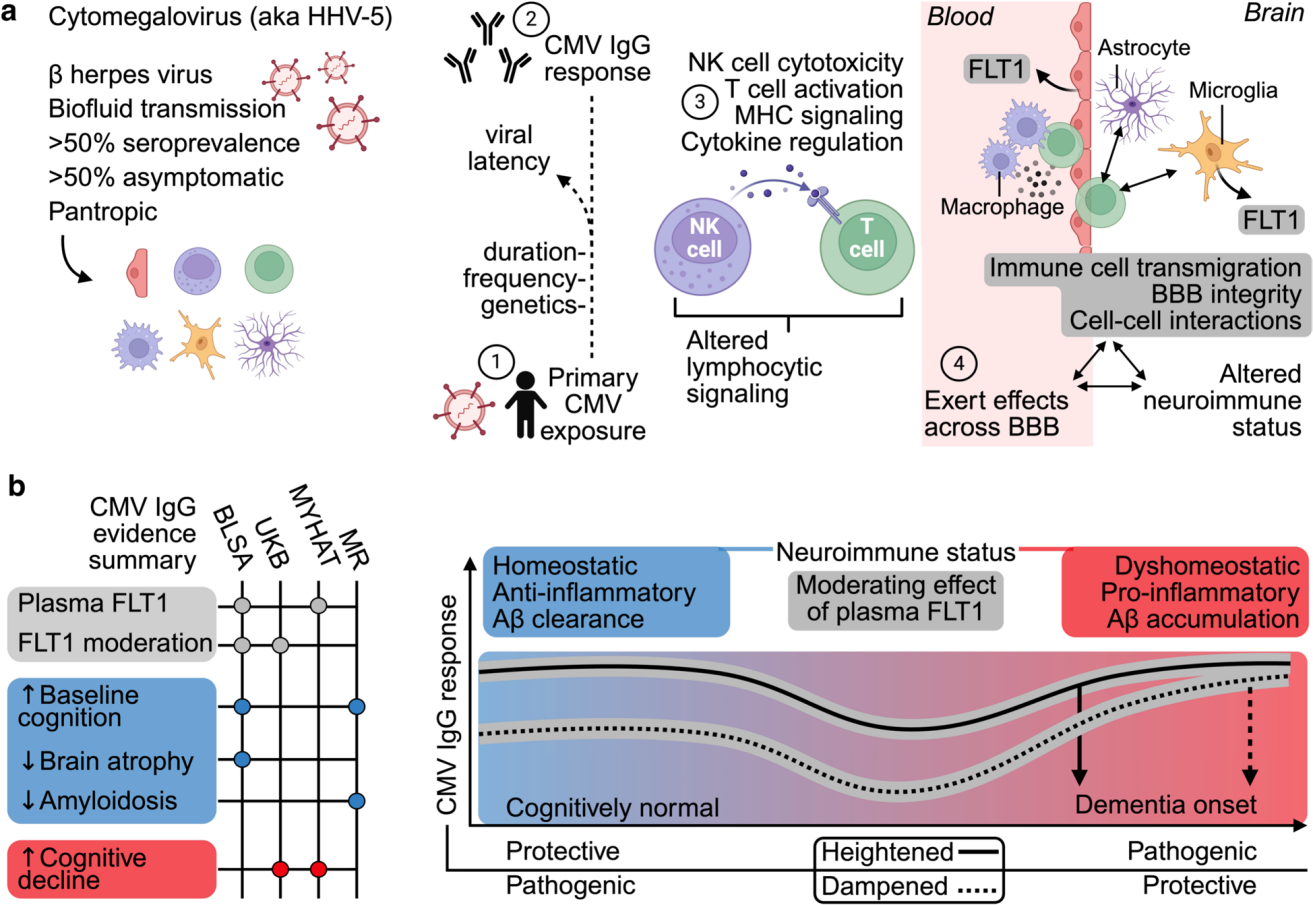
Conclusions and proposed model. **a** Potential mechanisms linking host immune responses to CMV, plasma FLT1 abundance, and neurologic health. CMV (cytomegalovirus) exposure via biofluids is relatively common, typically occurs in early life, and is often asymptotic, despite its capacity for infecting an array of cell types. In addition to neurological outcomes, CMV antibody (immunoglobulin G) responses are associated alterations in lymphocytic immunoregulatory cascades, including natural killer (NK) cell cytotoxicity and T cell activation. Because plasma FLT1 abundance moderates CMV’s associations with neurological outcomes and reflects alterations in immune cell transmigration, blood–brain-barrier (BBB) integrity, and cell–cell interactions, these results suggest circulating FLT1 abundance captures the extent to which CMV’s effects on lymphocytic immunoregulatory cascades can be transmitted across the BBB. **b** Evidence from the current investigation suggests pleiotropic associations of CMV titers with Alzheimer’s disease (AD) and related dementias (ADRD), which can be moderated by plasma FLT1 abundance. As suggested by prior biofluid and PET studies, the current findings indicate a biphasic pattern of immune processes in ADRD, whereby heightened immune responses may exert benefits during pre-clinical or prodromal stages of neurodegeneration, but also induce consequences with more advanced pathological burden

While these results imply that host immune responses to CMV may impact cognitive functioning through alterations in lymphocytic immunoregulatory cascades (e.g., NK cell mediated cytotoxicity), they also suggest such effects may be moderated by their capacity to transverse the BBB (e.g., via immune cell transmigration), which can otherwise be reflected in circulating FLT1 abundance (Fig. 5a).

## Discussion

Using data derived from MYHAT, BLSA, GNPC, and UKB, the current study identified how CMV antibody levels were associated with cognitive function and dementia risk in older adults, and leveraged plasma proteomics to elucidate potential biological mechanisms that may account for these relationships. Epidemiological evidence revealed pleiotropic associations of CMV titers with cognition, namely higher cross-sectional cognitive functioning (BLSA) and greater risk of cognitive decline (MYHAT, UKB). Additionally, we found genetic evidence supporting causal, protective roles for host immune responses to CMV, including for limiting brain amyloidosis. Plasma FLT1 (which is predominantly expressed in microglia and endothelial cells) was related to CMV titer levels (MYHAT, BLSA) and moderated CMV titer associations with cognition (BLSA, UKB). Employing multi-cohort (BLSA, UKB) plasma proteomic signatures of CMV titers and plasma FLT1, our bioinformatic analyses suggested that host CMV immune responses may impact neurologic health through alterations in lymphocytic immunoregulatory cascades, whereas circulating FLT1 abundance may capture the extent to which these effects can be transmitted across the blood–brain-barrier, particularly through cellular transmigration. Along with detecting pleiotropic associations of CMV titers with neurocognitive outcomes, these findings identify FLT1 as an important molecular moderator of these effects and extend our understanding of the biological processes by which host immune responses to common microbes may contribute to ADRD.

The associations of CMV titers with cognition in our study add to existing serological investigations of more common, often asymptomatic infections that have yielded similarly mixed results. CMV titers have been associated with cognitive impairment in some instances, but we recently found lower odds of dementia among participants with elevated CMV IgM levels, and others have reported positive correlations of CMV IgG titers with cognitive performance [8, 53, 54]. With many investigations reporting null associations, a comprehensive meta-analysis did not find a clear relationship between CMV antibody levels and ADRD [55]. The cross-sectional, protective associations of CMV titers observed in our study could indicate that a more robust immune response – manifested here against common microbes – initially renders some individuals more resistant to neurodegeneration (Fig. 5b). This interpretation is supported by our two sample MR results, which demonstrated causal roles for host immune responses to CMV in persevering cognitive performance and limiting amyloidosis. This interpretation is also supported by reports of elevated ADRD risk among individuals who are vulnerable to symptomatic infections, with vaccination against common microbes having the opposite effect [7, 56]. However, the longitudinal, pathogenic associations of CMV titers we observed could indicate that such a heightened immune response against common microbes confers an inverse effect over time (Fig. 5b). This biphasic postulation is supported by biofluid (e.g., TREM2, IL-12) and PET (e.g., TSPO, deprenyl) evidence suggesting that heightened immune responses can exert benefits during pre-clinical or prodromal stages of neurodegeneration, but also induce deleterious effects with more advanced pathological burden [2–5, 57].

While we interpret the coexistence of protective and deleterious associations across cohorts through a biphasic framework, we recognize that the cross-sectional (BLSA) and longitudinal (MYHAT, UKB) analyses were conducted in distinct populations, and therefore population heterogeneity may represent an equally plausible explanation for our results. Differences in baseline health status, vascular risk burden, systemic inflammatory profile, and degree of immunosenescence across cohorts may have influenced what elevated CMV IgG titers biologically represent in these different groups of participants. In relatively healthier or less immunosenescent individuals, higher titers may reflect preserved antiviral immune competence, but in populations with greater immune aging or comorbidity burden, high titers may more strongly reflect chronic immune activation, immunosenescence, and repeated viral reactivation [58–61]. Thus, the biphasic pattern we observed across studies may reflect within-individual, stage-dependent shifts and/or between-cohort differences in the dominant biological meaning of IgG titers. Building on these observations, future studies could benefit from incorporating both CSF and plasma markers of neuroinflammation (e.g., cytokine and chemokine panels) alongside CMV- or other pathogen-specific antibody titers, with nested sub-studies additionally examining longitudinal changes in immune cell phenotypes, endothelial activation, and viral reactivation events. Together, these data would help disentangle the associations of CMV titers with prevalent and incident neurocognitive outcomes.

The current proteomic and bioinformatic results are consistent with prior studies linking CMV titers to variation in lymphocytic biological processes, but we build on existing evidence by suggesting that such variation may also be relevant for cognitive functioning among older adults [62, 63]. Accumulating data indicate BBB permeability and adaptive immune cells play important roles in ADRD, including the ability of lymphocytes to perturb CNS homeostasis while residing in systemic circulation and contribute to pathophysiology following infiltration into the perivascular space, leptomeninges, and parenchyma [64–67]. FLT1 can directly recruit monocytes and lymphocytes to different endothelial locations through signaling with its canonical ligand, VEGF, and induce the upregulation of adhesion molecules (e.g., ICAM1, VCAM1) that are essential for leukocyte adhesion and diapedesis across the BBB. Along with its multi-cohort proteo-biological signature consistently enriched with immunological proteins, BBB biomarkers, and mechanisms underlying cellular transmigration (e.g., chemotaxis, chemoattraction, cell migration, cell motility), such existing literature supports our working hypothesis that circulating FLT1 abundance captures the extent to which CMV’s effects on lymphocytic immunoregulatory cascades and brain health can be transmitted across the BBB.

Along with these additions to existing literature, our two sample MR results demonstrating an inverse relationship between cortical Aβ deposition and CMV titers could indicate that the antimicrobial properties of Aβ documented for other herpetic viruses may also extend to CMV [68]. Despite genetic evidence supporting causal, protective roles for host immune responses to CMV with respect to limiting Aβ and preserving GCP (i.e., general cognitive performance), we did not detect associations with AD or ACD. It is possible this inconsistency reflects true biological discrepancy as well as differences in disease stage, phenotype definition, and/or statistical power across outcomes. Whereas AD and ACD are late-stage, clinically defined syndromes that represent heterogeneous and multifactorial endpoints, GCP and cortical Aβ can represent earlier or more proximal phenotypes along the neurodegenerative continuum that may be more sensitive to subtle, cumulative biological effects. It is therefore plausible that CMV-related immune variation influences intermediate endophenotypes—such as Aβ accumulation or cognitive efficiency—without translating into a detectable causal effects on clinically defined AD or ACD in the available GWAS datasets. Supporting this notion is the fairly common existence of age-related cognitive decline and cortical Aβ among individuals without clinically defined dementia, and the underlying genetic architecture for these two intermediate phenotypes which is not fully overlapping with that of dementia [69].

CMV titers were positively associated with concurrently measured FLT1 in the BLSA and negatively associated with future FLT1 levels in MYHAT, and our analyses suggested that different inflammatory profiles between BLSA and MYHAT proteomic data (but not other cohort differences like demographics or timing of blood draws) may have contributed to these discordant FLT1-CMV associations. One potential reason for such differing inflammatory profiles is that MYHAT proteomic data were derived from blood draws collected from an older subset of participants who provided a second blood sample approximately 11.0 years (on average) after CMV titers were measured, whereas BLSA proteomic and titer data were derived from the sample blood draw. Although our results should be interpreted with caution because they only examined a subset of the broader immunological proteome, they suggest that the enhanced cognitive resiliency observed among BLSA participants with higher CMV antibody responses and higher FLT1 abundance may be partially attributed to a broader, attenuated inflammatory mileu. Discrepancies may also be due to cross platform differences in the measurements of FLT1 between cohorts. Specifically, the cross-platform correlation of FLT1 measurements between NULISA and SomaScan is modest (*rho* = 0.34), suggesting these assays may capture heterogeneous measurements and reflect partially distinct aspects of circulating FLT1 biology (e.g., isoform specificity, epitope recognition, dynamic range).

Given that FLT1 levels augmented the protective associations of CMV titers with prevalent dementia in the BLSA and exacerbated the deleterious associations of CMV titers with incident dementia in the UKB, it is possible FLT1’s biological functions remain consistent throughout older ages and disease stages, but that such functions act synergistically with the known biphasic neuroinflammatory trajectories that accompany neurodegenerative disease progression [2–5, 57]. Early on in the disease course, we hypothesize that individuals with robust adaptive immune responses to CMV also illustrate homeostatic FTL1 signaling with its high affinity ligand, VEGF [49]. Along with promoting tissue perfusion through angiogenesis, endothelial cell repair, and vascular integrity, this balanced FLT1-VEGF signaling promotes recruitment of immune cells while also inducing the upregulation of adhesion molecules that are essential for cellular transmigration across the BBB [70–76]. Under normal physiological conditions, such FLT1-dependent enhancement of neurovascular permeability and immune cell-migration may support brain health and boost the hypothesized benefits of immune activation early on in the course of neurodegeneration [77, 78]. As disease course progresses and inflammation becomes more chronic, sustained VEGF production and/or altered FLT1 expression may disrupt this balance. Pathological conditions and pro-inflammatory states can trigger a shift in FLT1’s soluble versus membrane-bound ratios, which could enhance decoy receptor binding, aberrantly sequester VEGF, and impair endothelial survival [79, 80]. Prolonged FLT1-VEGF signaling in response to neuroinflammation can increase paracellular permeability in the neurovascular unit by downregulating transmembrane tight junction proteins (e.g., claudin-5, occludin) [81]. Meanwhile, FLT1's similar recruitment of immune cells during advanced disease stages (i.e., when aberrant inflammatory cascades have already been initiated) may perpetuate a vicious cycle of abnormal neuroinflammation that is associated with a net detrimental effects [82]. Thus, under pathological conditions, such FLT1-dependent signaling may compromise neurovascular integrity and promote aberrant immune cell-migration that exacerbates the hypothesized consequences of immune activation later on in the course of neurodegenerative disease. In this model, FLT1 does not intrinsically change function; rather, its biological output shifts due to changes in abundance, altered binding profiles, and inflammatory context. We therefore propose that FLT1 acts as an immunovascular modulator whose net effect transitions from protective to pathogenic as the inflammatory milieu evolves. Longitudinal measurement of FLT1/VEGF ratios, single-cell profiling of FLT1-expressing populations, conditional knockdown and overexpression of FLT1 under normal and pathological conditions, and isoform-specific functional perturbation studies would provide mechanistic validation of this stage-dependent model.

The present findings offer opportunities for multifaceted, broader impacts. As our study suggests that in some contexts the robustness of an individual’s immune response to common infections may be a risk or protective factor for neurodegenerative disease, future work should continue to investigate these immunological mechanisms as viable treatment targets. Vaccination is one obvious intervention with a potentially high public health impact, but the repurposing of other FDA approved treatments should also be considered. Specifically, our findings suggest the repurposing of FDA approved treatments which modulate lymphocytic pathways (e.g., ciclosporin that prevents T cell activation and clonal expansion), BBB integrity (e.g., dexamethasone that reduces inflammation and BBB permeability), and immune cell transmigration (e.g., natalizumab that inhibits leukocyte trafficking into the CNS) could offer benefits for older individuals at risk for dementia [83–86]. In light of the observed pleiotropic associations with CMV titers, we cannot speculate at what disease stage these medications would be most efficacious, nor can we infer if antiviral and antiherpetic therapeutic interventions would be beneficial. Moreover, these results reflect an addition to the growing body of knowledge that advances our understanding of the dynamic relationships between microbes, immune functioning, and neurodegeneration, an area of investigation that has been chronically understudied and underfunded [87]. Given the growing evidence of heightened ADRD risk following severe infections, the increasing number of older adults at risk for developing neurological diseases, and the increasing number of individuals exposed to different microbes, the current landscape could substantially benefit from further investigations that deconvolute biological mechanisms by which host defenses against common microbes can contribute to differing neurological outcomes.

Interestingly, in the UKB, pp28 IgGs were linked to differential AD risk, but pp52 and 150 titers were not. Although these are all viral products of CMV, they differ substantially in their biological functions, immunogenicity, and temporal expression patterns during the viral life cycle [88, 89]. pp28 (encoded by *UL99*) is a late structural tegument protein involved in cytoplasmic virion assembly and secondary envelopment. Antibody responses to late structural viral proteins may more directly reflect cumulative viral burden, viral reactivation frequency, or the efficiency of viral assembly and release. In contrast, pp52 (encoded by *UL44*) is an early non-structural DNA polymerase processivity factor expressed during viral replication in the nucleus, and pp150 (encoded by *UL32*) is an intermediate capsid-associated tegument protein; antibody responses to these proteins may reflect different phases of viral biology which may not capture the same aspects of chronic immune stimulation. Thus, these results could indicate that antibody responses to cumulative viral burden or repeated viral reactivation (i.e., which is better reflected in pp28 titers than pp52 and/or pp150 titers) may be particularly important contributors to incident AD.

When inferring the implications of the current results, an important conceptual consideration is the interpretation of CMV IgG antibody titers as a proxy for host immune response to CMV. A single-point IgG measurement is a static biomarker that may reflect at least two partially overlapping biological processes. First, higher titers may represent a trait-like marker of robust humoral immune responsiveness, reflecting preserved immune competence and the capacity to maintain effective antiviral surveillance. Second, elevated titers may indicate chronic or recurrent antigenic stimulation due to viral reactivation, reflecting a state of persistent immune activation and cumulative antigenic burden. These interpretations are not mutually exclusive and may vary according to age, degree of immunosenescence, comorbidity burden, and disease stage. Importantly, a single IgG measurement cannot distinguish between steady-state immune competence and ongoing viral reactivation. Future studies incorporating complementary markers — such as IgG avidity indices, CMV IgM or DNAemia to detect recent reactivation, and CMV-specific T-cell phenotyping to assess functional immune profiles — will be essential to disentangle these distinct biological states and refine mechanistic inferences derived from the present findings.

Our study has several strengths, including multi-cohort serology from population- and community-dwelling participants, state-of-the-art proteomics, orthogonal strategies for assessing cognition (i.e., neuropsychological testing, dementia adjudication, etiology-specific dementia risk), and genetic techniques to support causality. However, several limitations warrant consideration. First, because we focused on examining how host immune responses to specific microbes may be related to neurocognitive outcomes, we did not investigate the role of generalized immune responses against multiple microbes or immune signatures of the larger microbiome. Future studies should apply complementary techniques (e.g., serology, polymerase chain reaction, antigen tests, etc.,) and different biospecimens (e.g., blood, CSF, brain tissue etc.,) to pursue this line of inquiry with more reliable and comprehensive measures of pan-microbial abundance. Second, following FDR correction in MYHAT discovery analyses, several CMV titer associations with longitudinal cognitive task performance were not retained. Although analyses in external cohorts (BLSA, UKB) were used to reduce potential type 1 error and supported a potential biphasic relationship between CMV antibody levels and cognition, we acknowledge that associations with CMV titer levels across cohorts may be inconsistent or absent (i.e., as suggested by a recent meta-analyses [55]). Third, due to insufficient statistical power and sample diversity for some analyses (e.g., MYHAT proteomic analyses, *n* = 43; UKB dementia analyses, 5.3% non-white), we were unable to reliably interrogate whether our findings were modified by factors that can be related to both immune responses and age-related cognitive decline, such as gender, race, and socioeconomic status [90–92]. While we encourage future investigations to validate our findings unhindered by these limitations, the current study nonetheless provides insights for how host immune responses to CMV may be related to cognitive capacities among older adults, and identifies the biological basis that may account for these relationships.

## Supplementary Information

Supplementary Material 1.

Supplementary Material 2.

## Data Availability

MYHAT data used in the present study are available upon reasonable request. Requests can be made using the “Contact Us” link on the MYHAT public-facing website (https://www.dementia-epidemiology.pitt.edu/) and are subject to approval in accordance with data sharing policies at the University of Pittsburgh. All BLSA data generated in the present study are included in this article, available on reasonable request, or in an online public repository. BLSA proteomic data are available via the Alzheimer’s Disease Data Initiative (https://www.neuroproteome.org). Participants did not consent to unrestricted data sharing. Anonymized data not published within this article may be shared upon request from qualified investigators for purposes of replicating procedures and findings. Researchers who wish to use BLSA data (including proteomics) are encouraged to develop a pre-analysis plan that can be submitted for approval (https://blsa.nia.nih.gov/how-apply). Data, protocols, and other metadata of the UKB are available to the scientific community upon request in accordance with the UKB data sharing policy (https://www.ukbiobank.ac.uk/enable-your-research/apply-for-access). No original code was developed for this study, and no custom code or mathematical algorithm was central to its conclusions. Analyses used publicly available R packages, including stats (4.4), tidyverse (2.0), nlme (3.1), and ggplot2 (3.5), with basic functions (e.g., lm, full_join, geom_point etc.,).
