# Supplementary material for: CMV titer associations with cognition and the plasma proteome implicate FLT1 and neurovascular mechanisms as potential moderators
Source: J Neuroinflammation. 2026 Apr 16;23:169. doi: 10.1186/s12974-026-03800-8 (PMC13214193; doi:10.1186/s12974-026-03800-8)
Supplement: Supplementary file 1 — Supplementary Material 1. [file 12974_2026_3800_MOESM1_ESM.pdf]

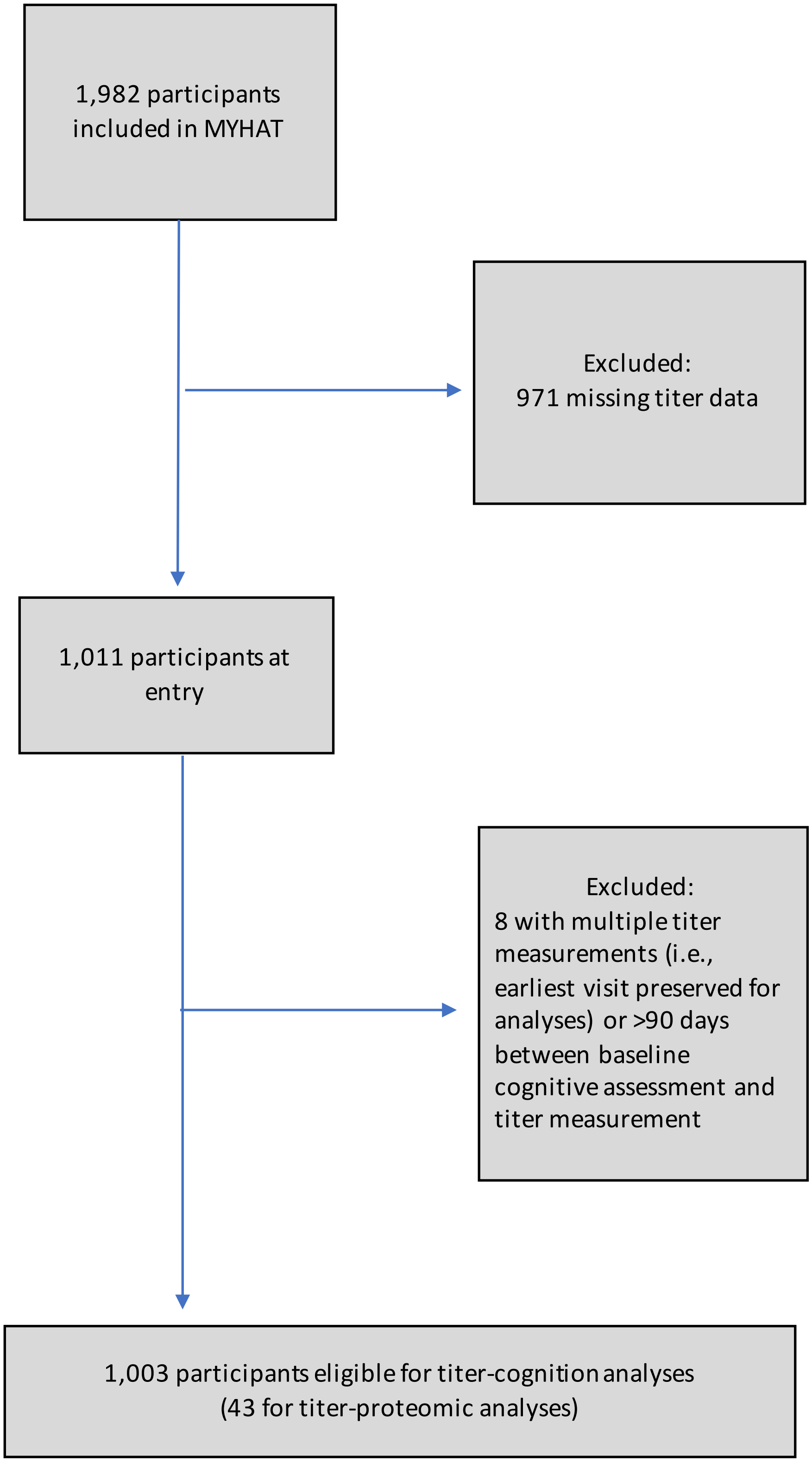

sFigure 1. Flow chart of MYHAT participant selection.

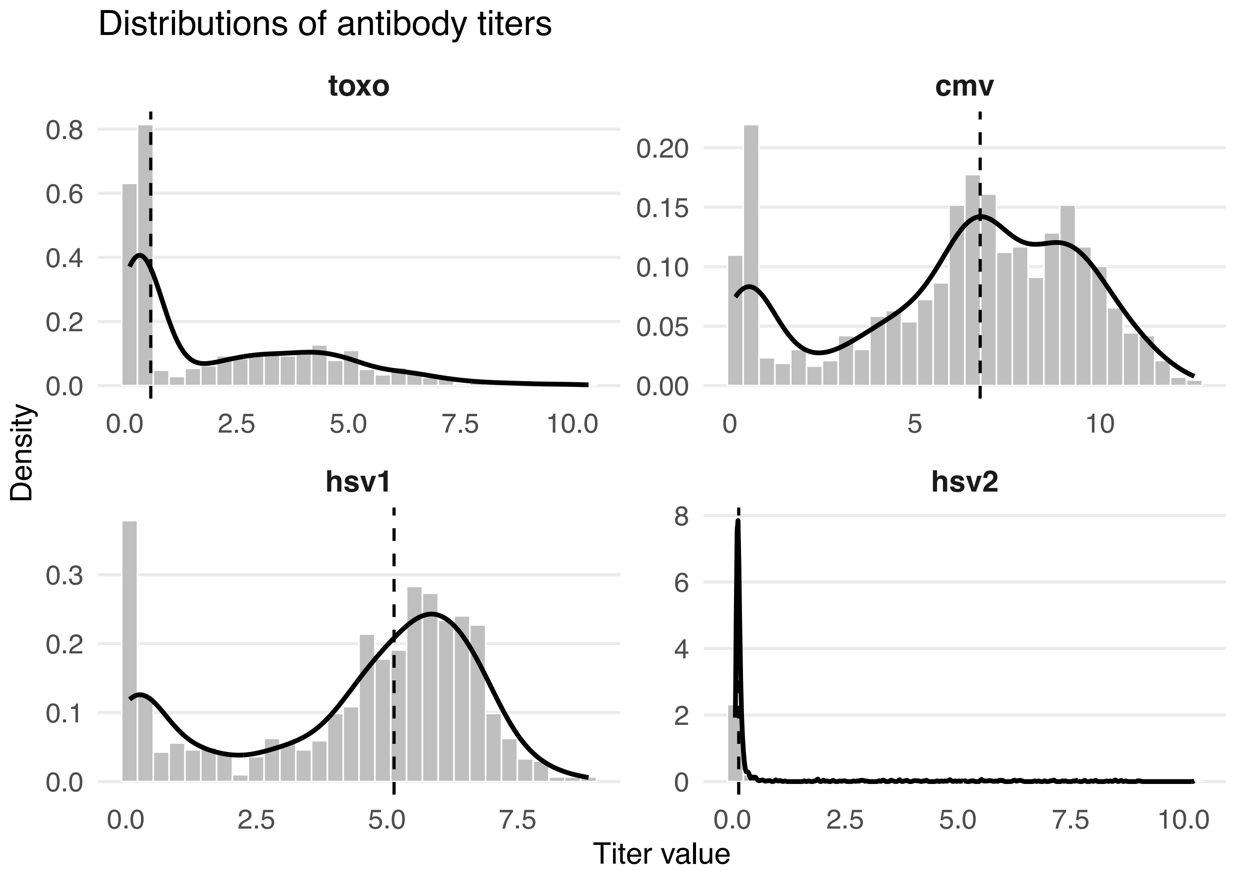

sFigure 2. Histograms shows the distributions of titer measurements in MYHAT. The dashed vertical lines reflect median distributions.

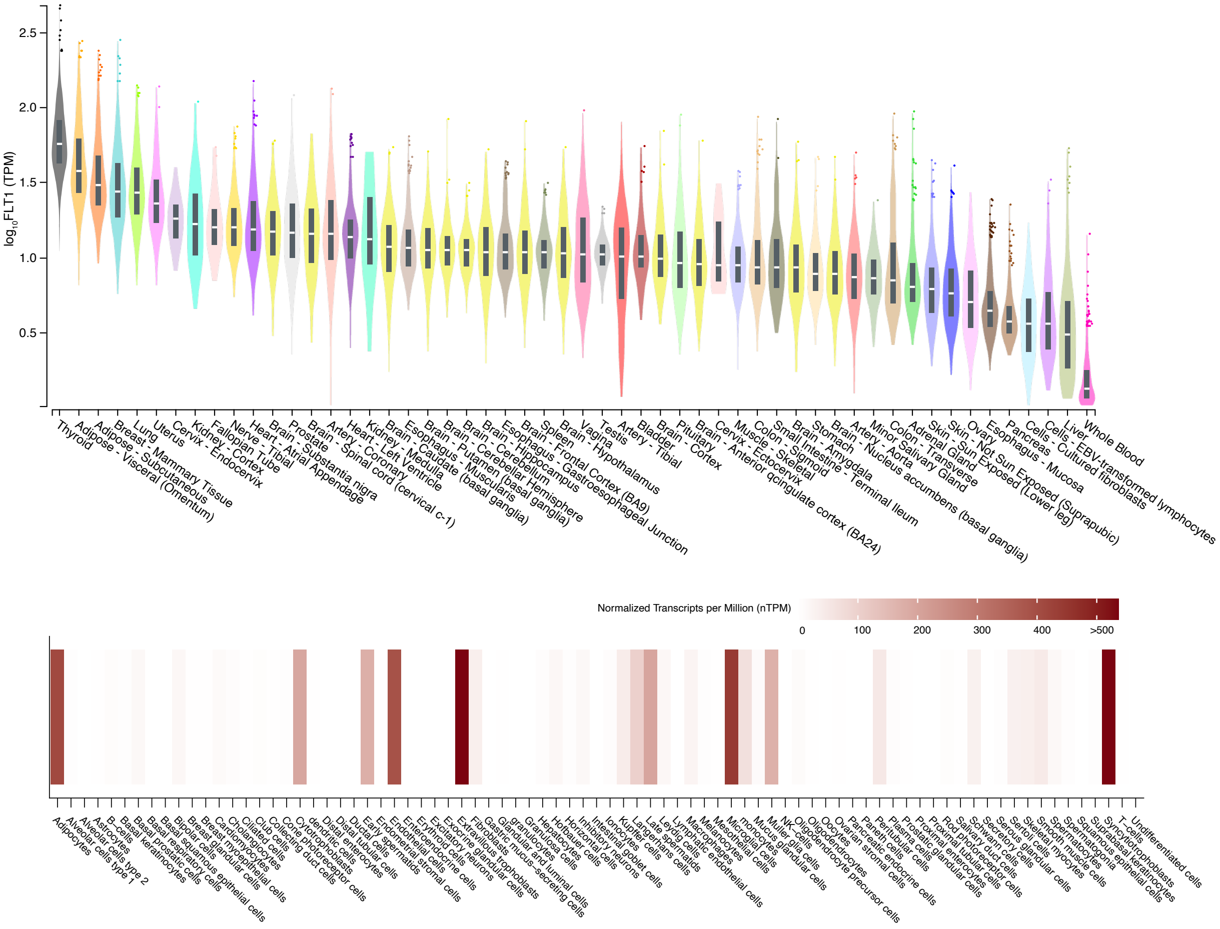

sFigure 3. FLT1 expression levels across tissue types and cell types based on bulk RNA sequencing from the Genotype-Tissue Expression project and Human Protein Atlas, respectively.

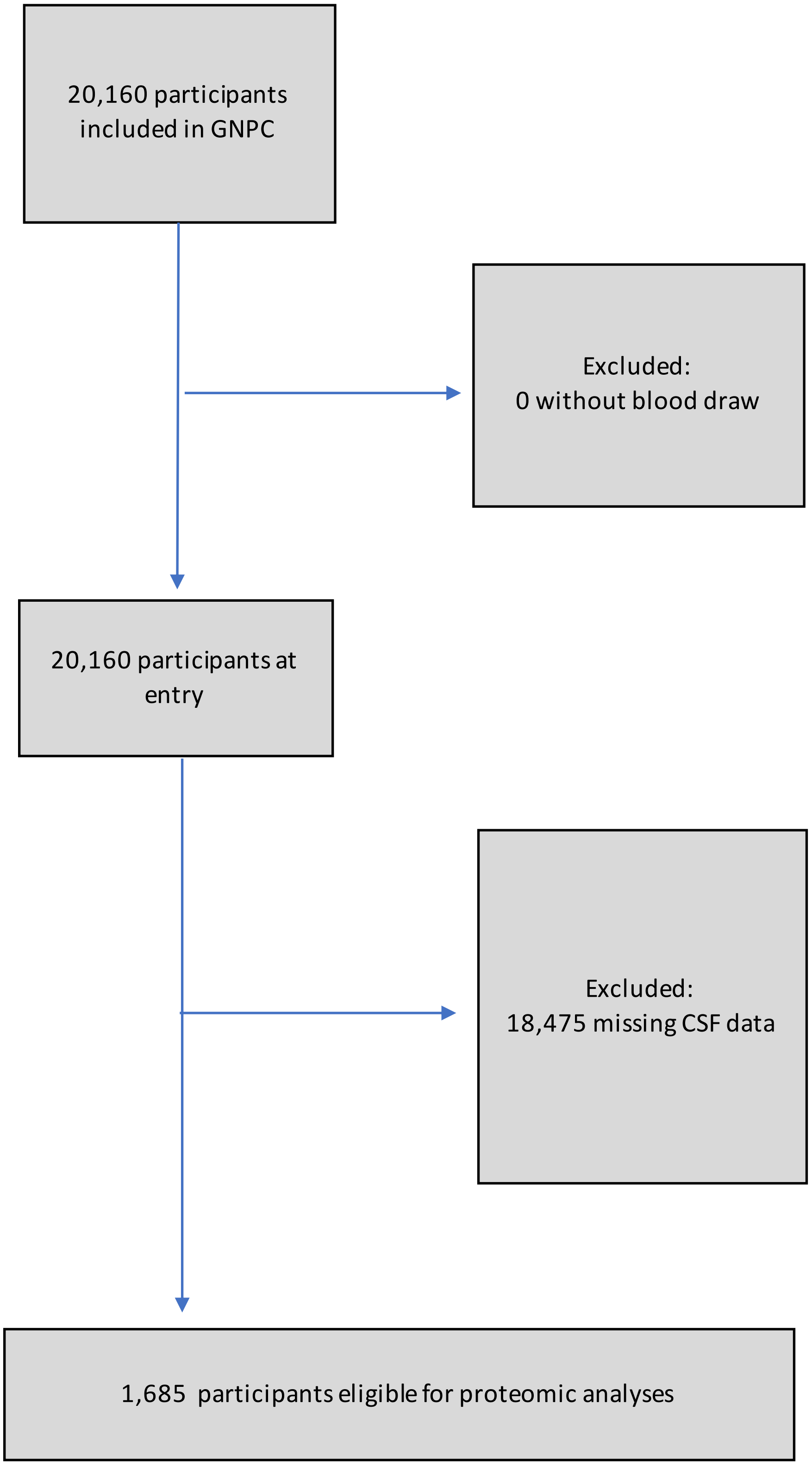

sFigure 4. Flow chart of GNPC participant selection.

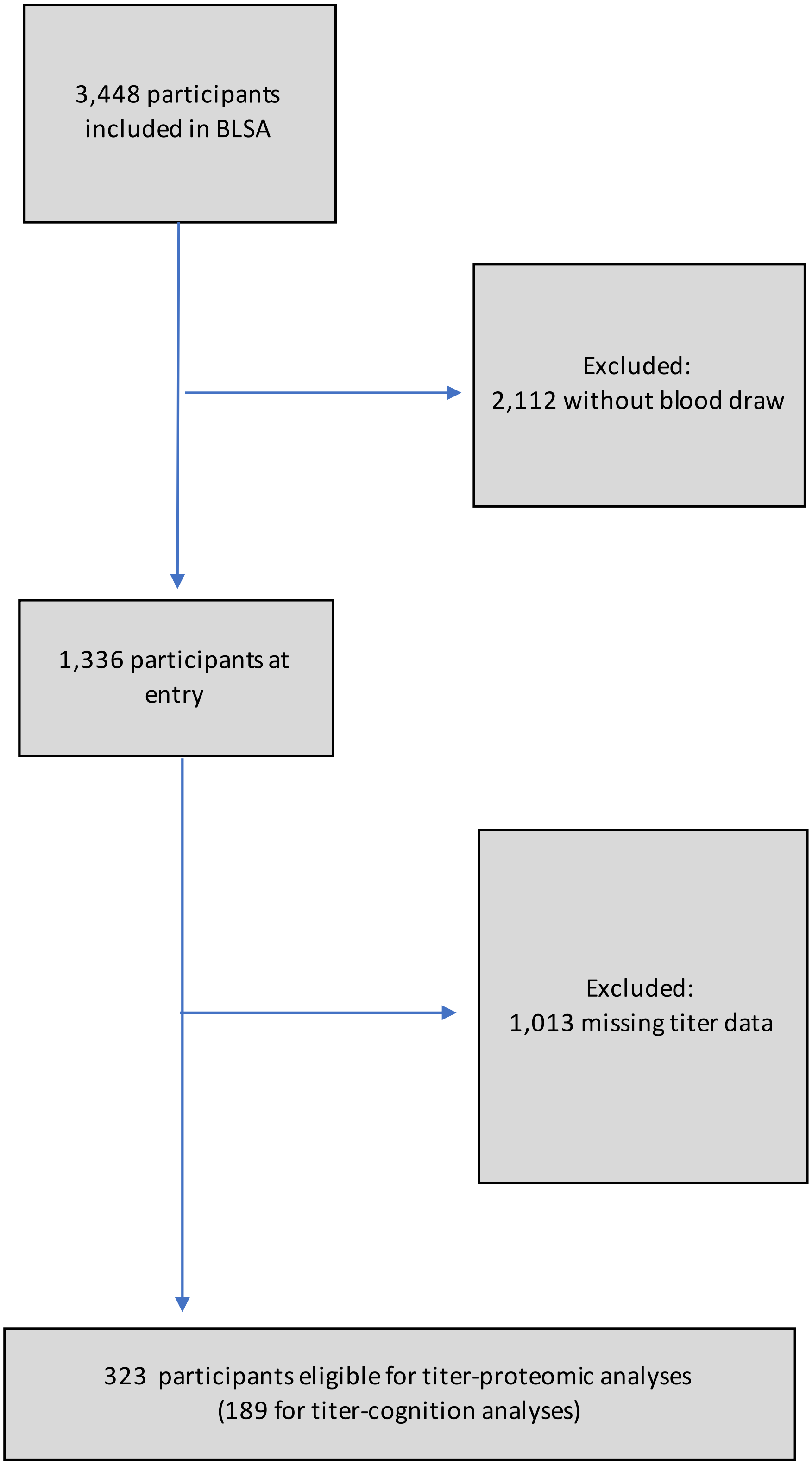

sFigure 5. Flow chart of BLSA participant selection.

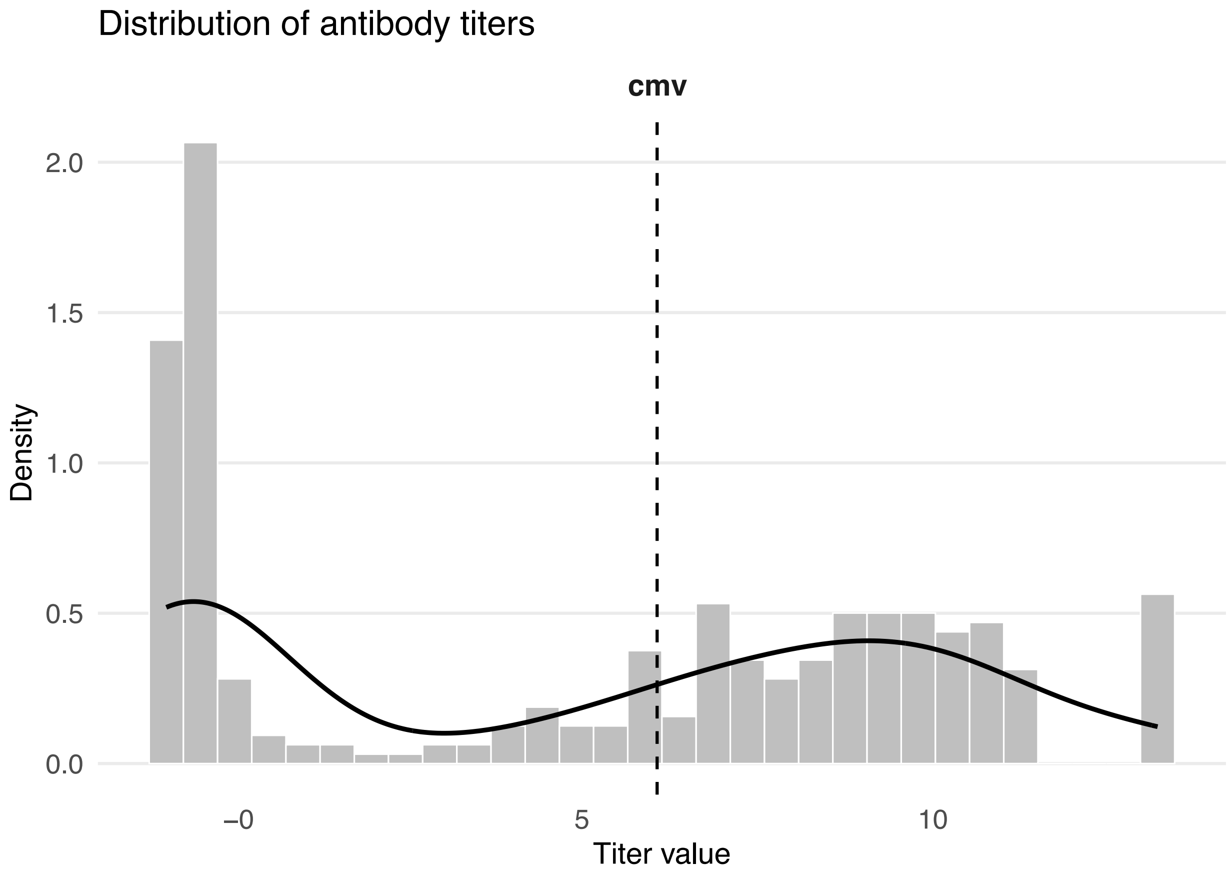

sFigure 6. Histogram shows the distributions of titer measurements in BLSA. The dashed vertical line reflects median distribution.

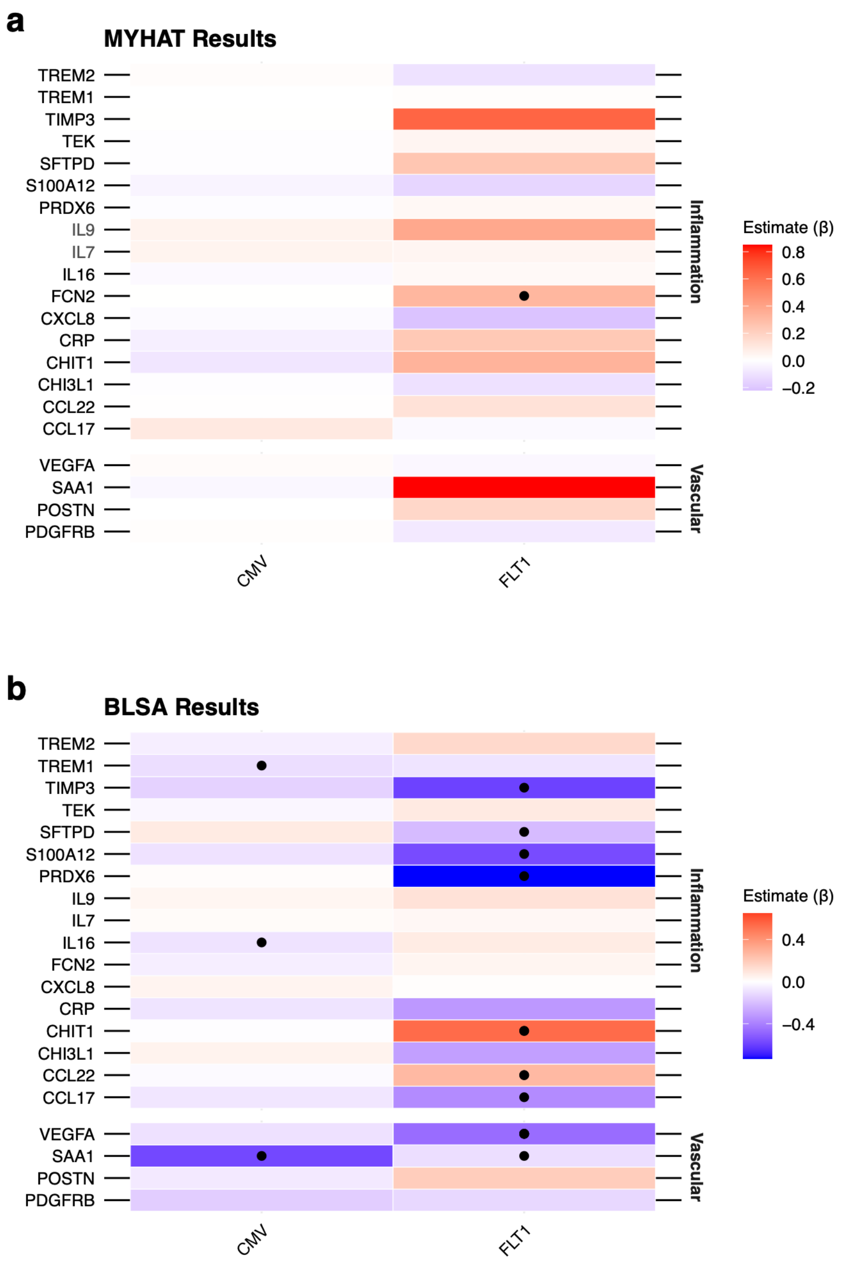

sFigure 7. CMV titer and plasma FLT1 associations with inflammation and vascular markers in MYHAT (**a**) and BLSA (**b**). Inflammation and vascular protein designations were identified through the NULISAseq CNS Disease Panel product information from the manufacturer, Alamar. Analyses were restricted to proteins with high correlations across platforms (i.e., *rho*≥0.50), as reported elsewhere (Ibanez et al., 2025; PMID: 39575871). Dots indicate significant associations (p<0.05). Results derived from linear regression models adjusted for age and sex.

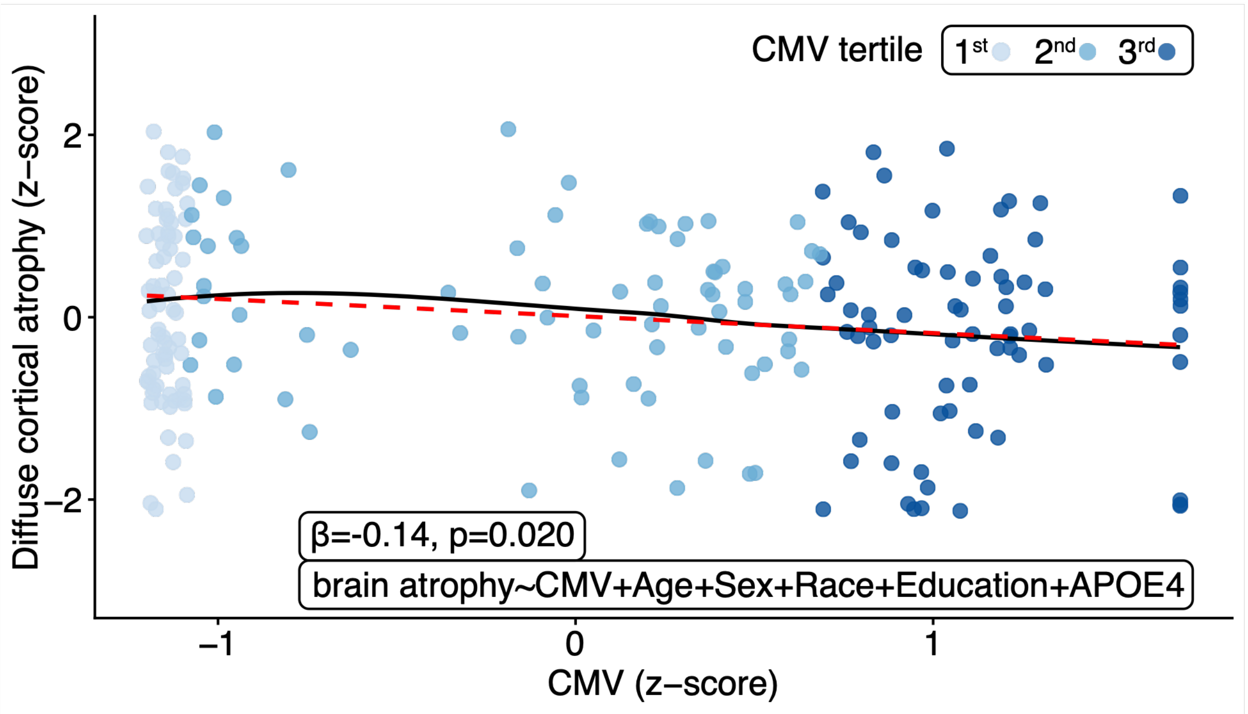

sFigure 8. Scatterplot of CMV titer associations with diffuse cortical atrophy measurement in the BLSA. Results derived from linear regression models adjusted for age, sex, race, education, and *APOEε*4.

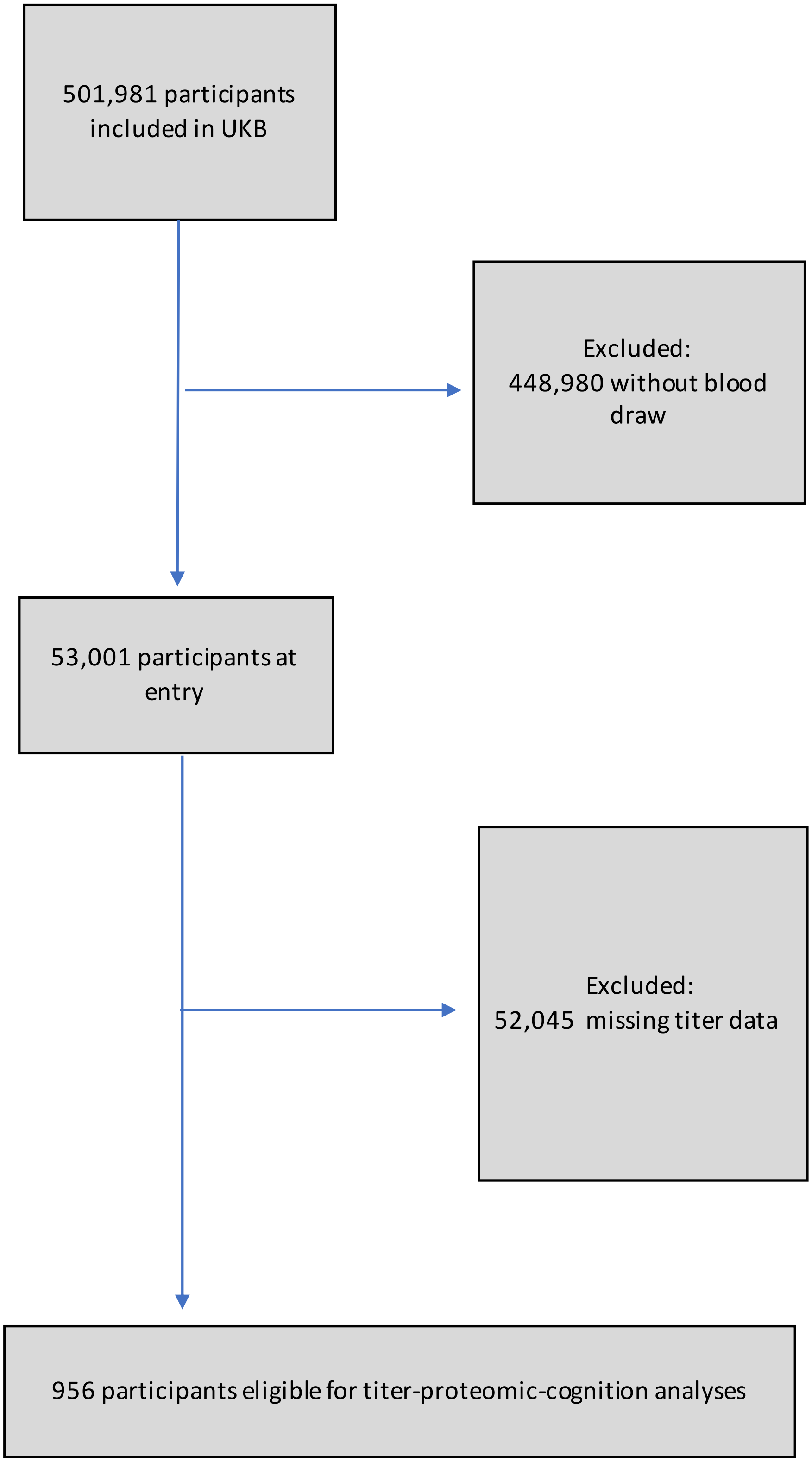

sFigure 9. Flow chart of UKB participant selection.

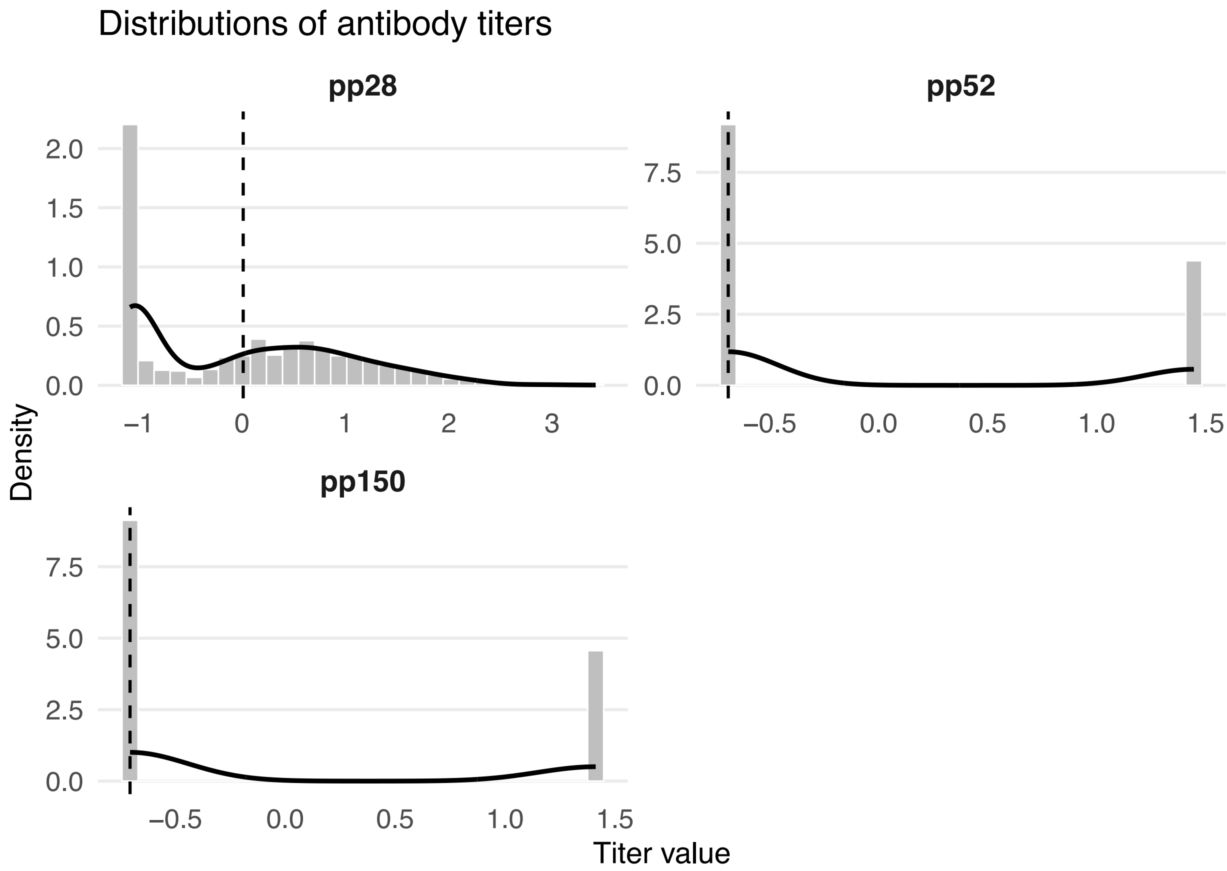

sFigure 10. Histogram shows the distributions of titer measurements in UKB. The dashed vertical lines reflect median distributions.

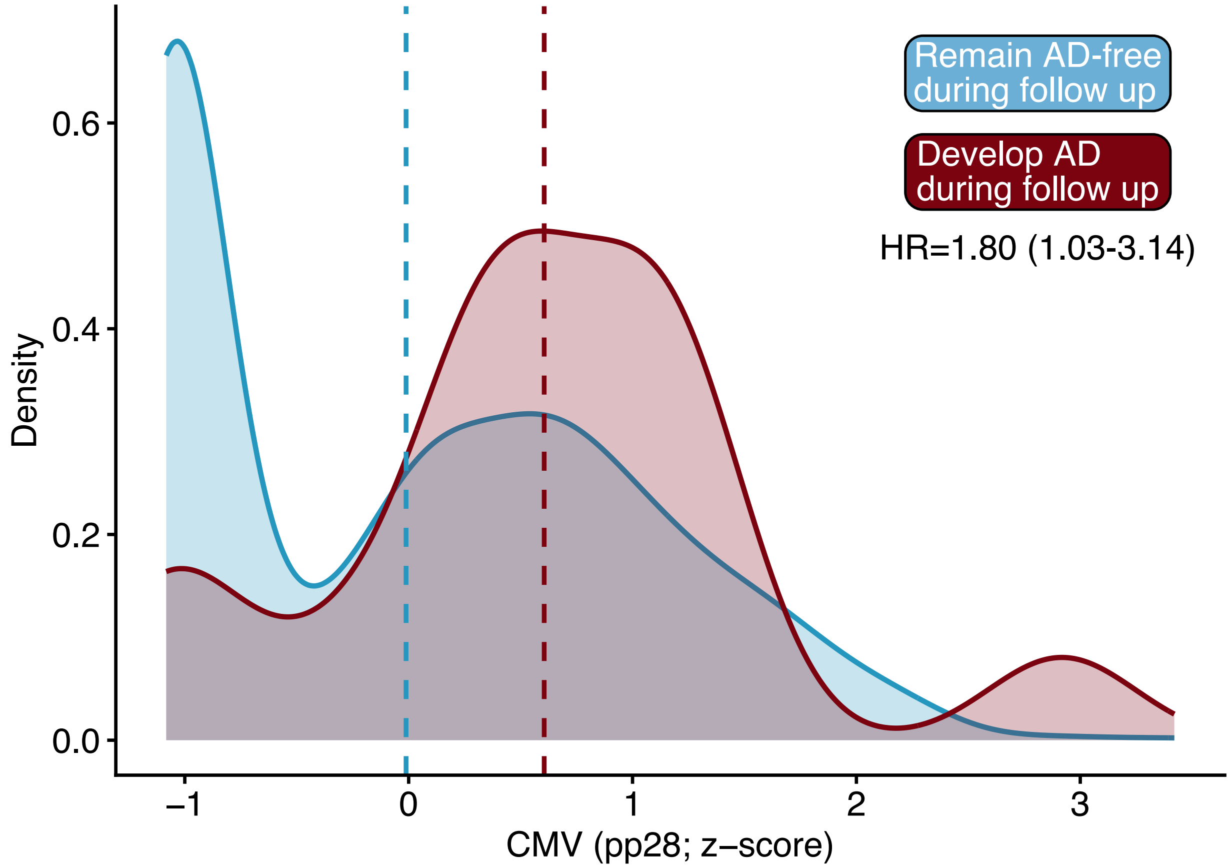

sFigure 11. Density plot of CMV titers (pp28) among individuals who do and do not develop Alzheimer's disease (AD) in UKB. Results derived from Cox regression models adjusted for age, sex, race, education, and *APOE*ε4.

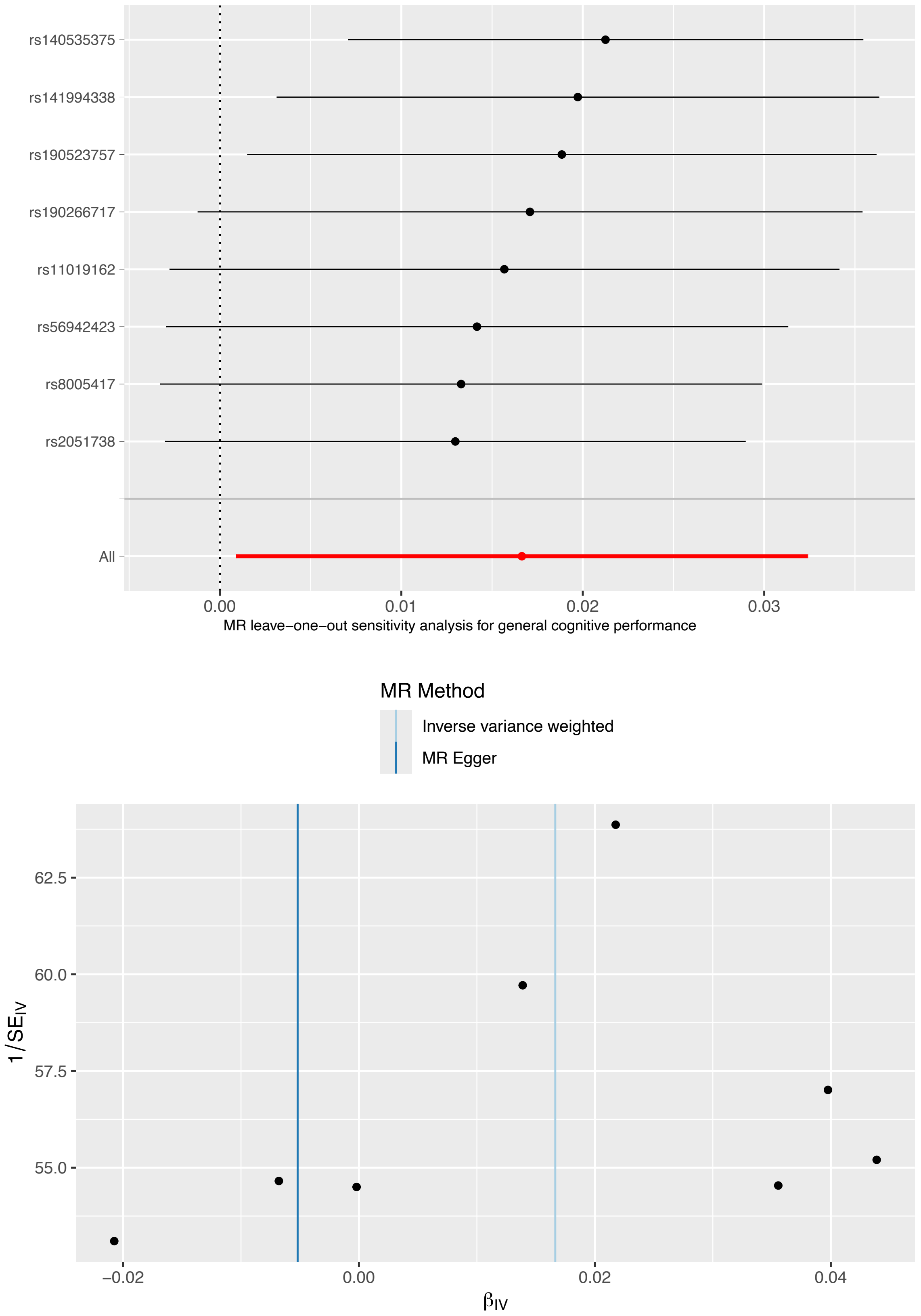

sFigure 12. Forest plot of leave-one-out sensitivity analyses and funnel plot of individual SNPs for two-sample MR with general cognitive performance as the outcome.

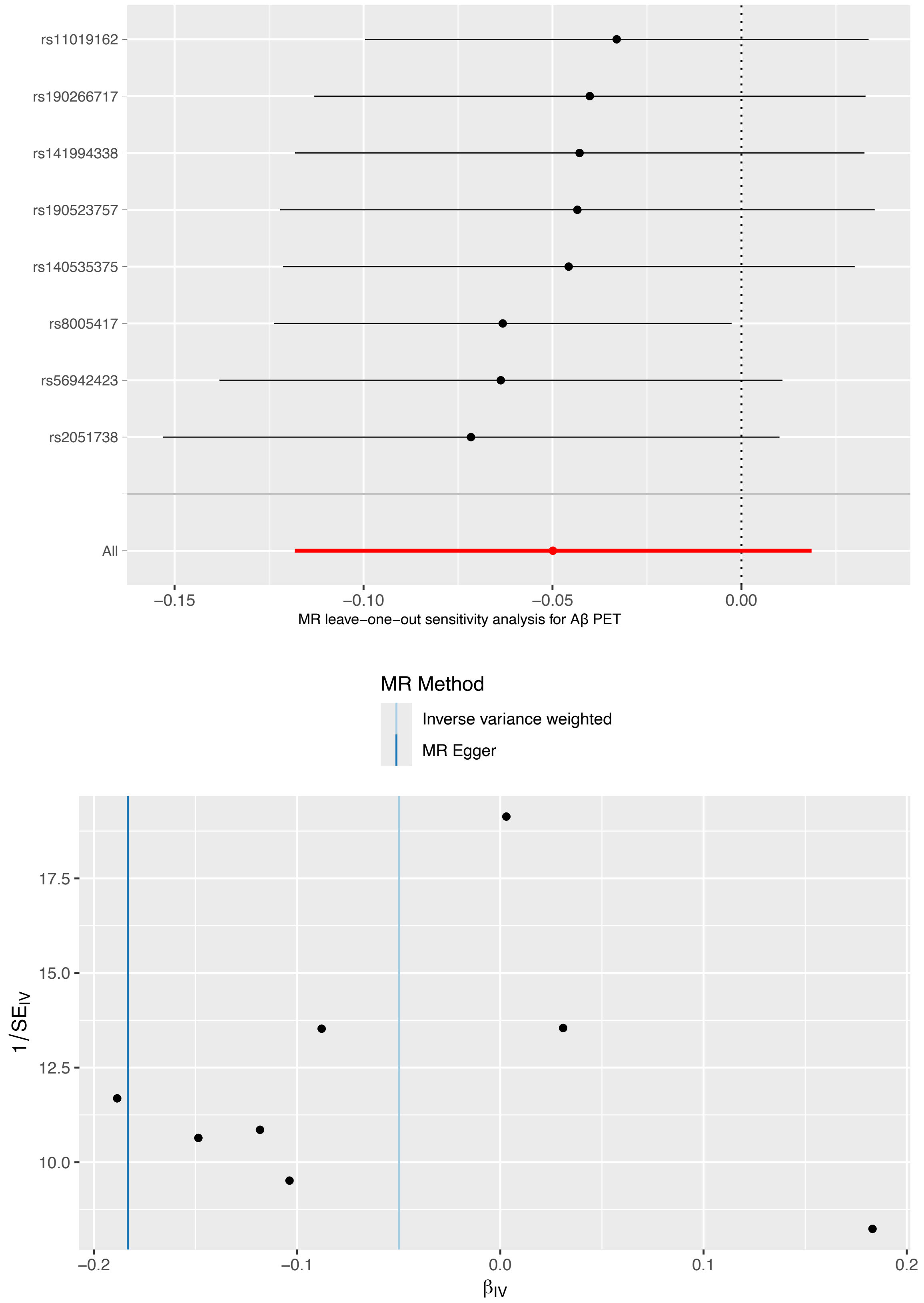

sFigure 13. Forest plot of leave-one-out sensitivity analyses and funnel plot of individual SNPs for two-sample MR with Aβ PET as the outcome.

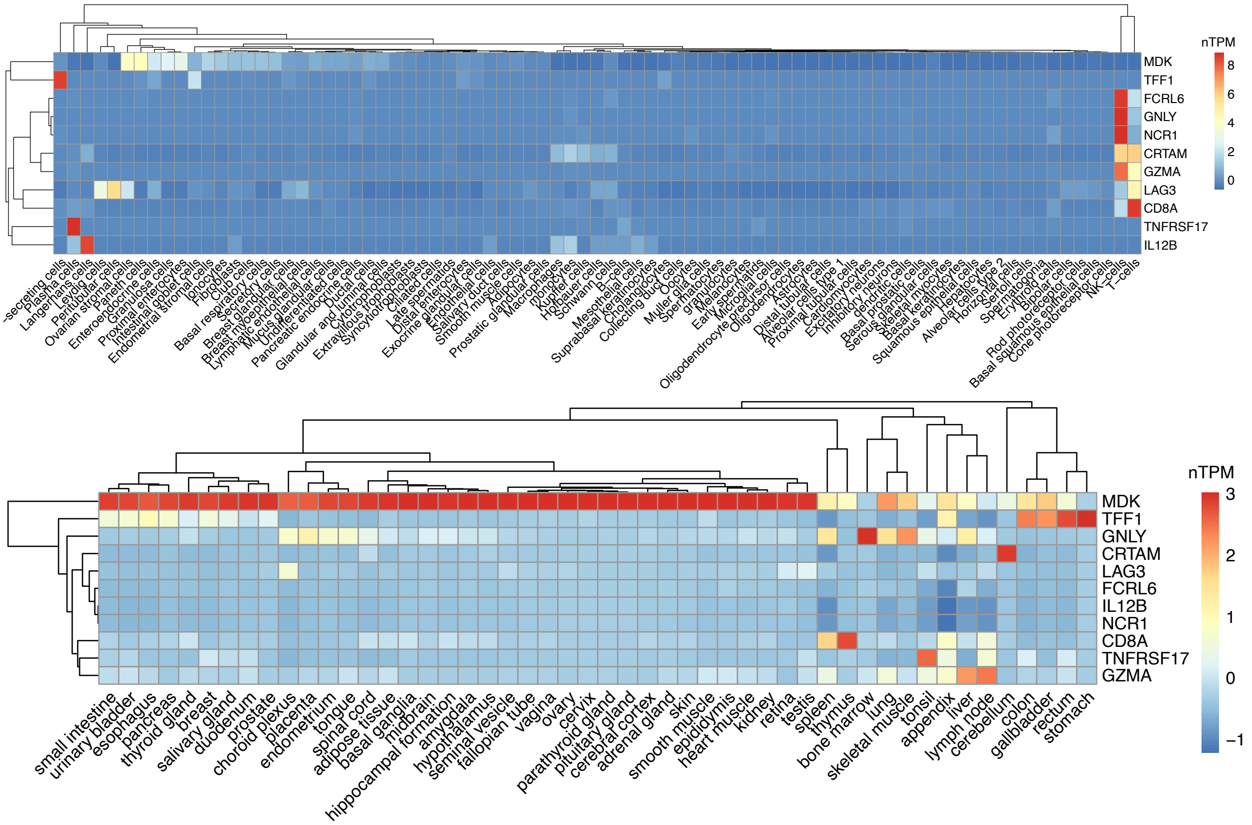

sFigure 14. Heatmaps show expression levels of genes encoding proteins that showed consistent directional associations with CMV titers across BLSA and UKB. Expression levels across 81 cell types and 50 tissue types were based on single cell transcriptomics and bulk RNA sequencing, respectively, sourced from the Human Protein Atlas. Dendrograms reflect hierarchical clustering using Euclidean distances calculated from normalized Transcripts per Million (nTPM). nTPMs used to generate heatmaps were standardized within each gene to improve interpretability. Key: nTPM, normalized Transcripts per Million.

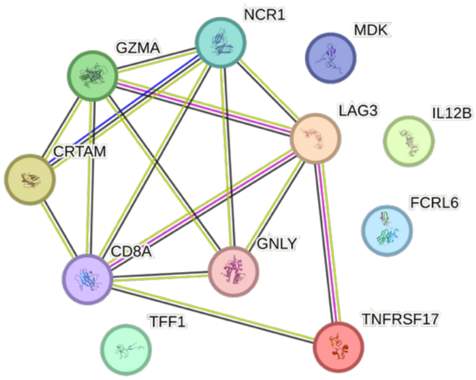

sFigure 15. Protein-protein interaction network of proteins that showed consistent directional associations with CMV titers across BLSA and UKB. Light blue lines indicate interactions derived from curated databases, and pink lines denote interactions that have been experimentally determined. Predicted interactions are shown using green lines for gene neighborhood, red lines for gene fusions, and blue lines for gene co-occurrence. Additional evidence channels include olive lines for text-mining support (co-mentioning in the scientific literature), black lines for co-expression evidence, and grey lines for protein homology. Results derived from STRING (Search Tool for the Retrieval of Interacting Genes/Proteins).

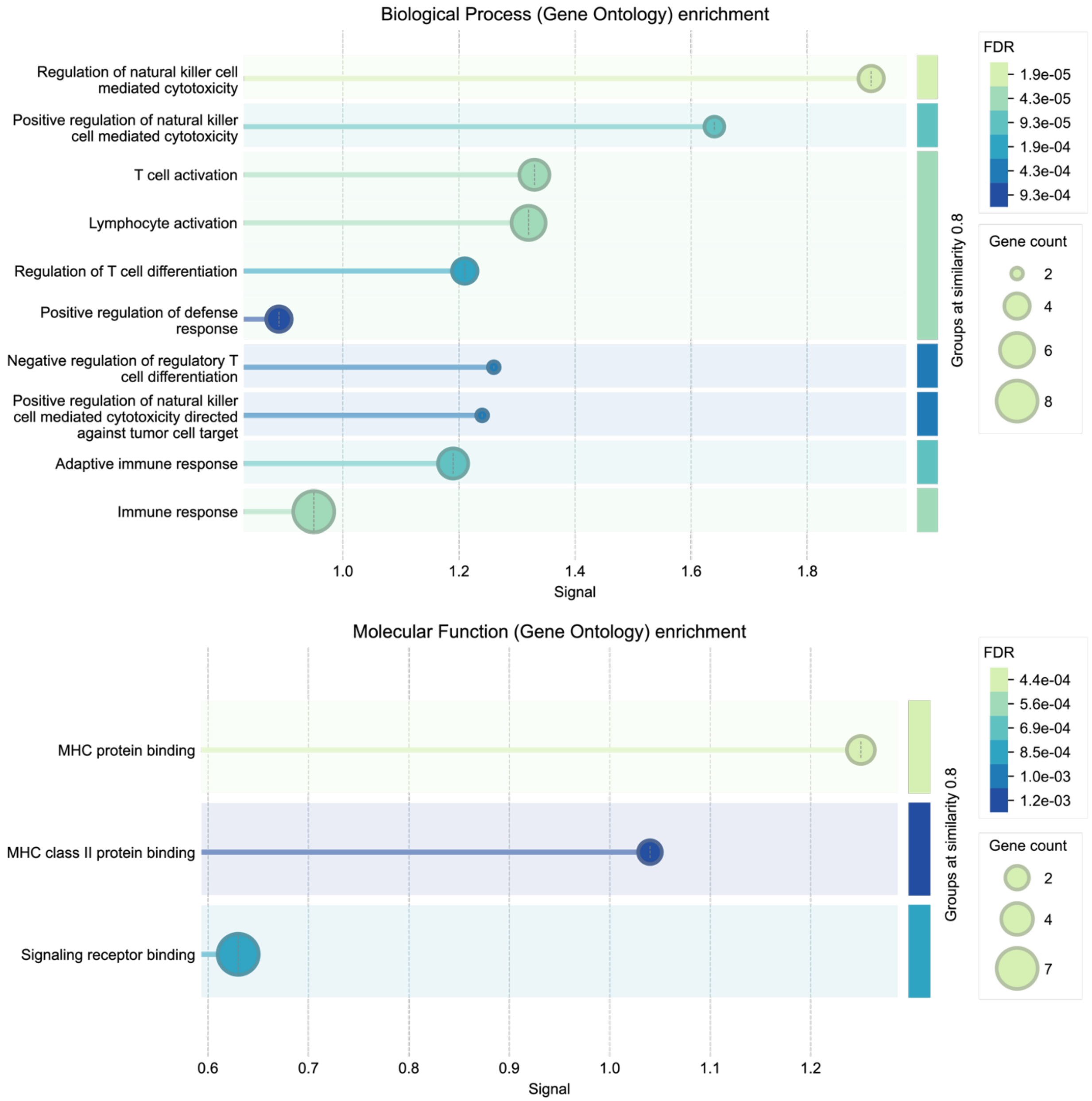

sFigure 16. Pathway enrichment (biological processes and molecular functions) of proteins that showed consistent directional associations with CMV titers across BLSA and UKB. Results derived from STRING (Search Tool for the Retrieval of Interacting Genes/Proteins).

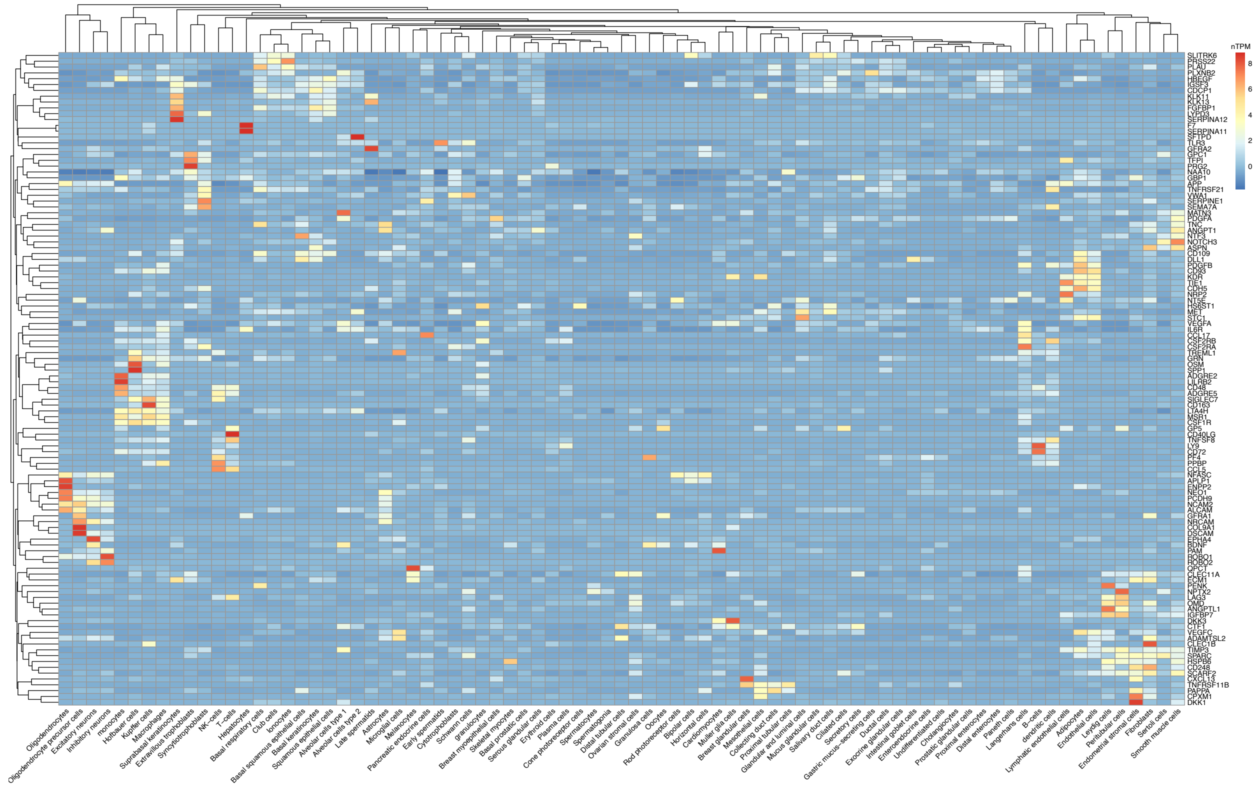

sFigure 17. Heatmap shows expression levels of genes encoding proteins that showed consistent directional associations with plasma FLT1 abundance across BLSA and UKB. Expression levels across 81 cell types were based on single cell transcriptomics sourced from the Human Protein Atlas. Dendrograms reflect hierarchical clustering using Euclidean distances calculated from normalized Transcripts per Million (nTPM). nTPMs used to generate heatmaps were standardized within each gene to improve interpretability. Key: nTPM, normalized Transcripts per Million.

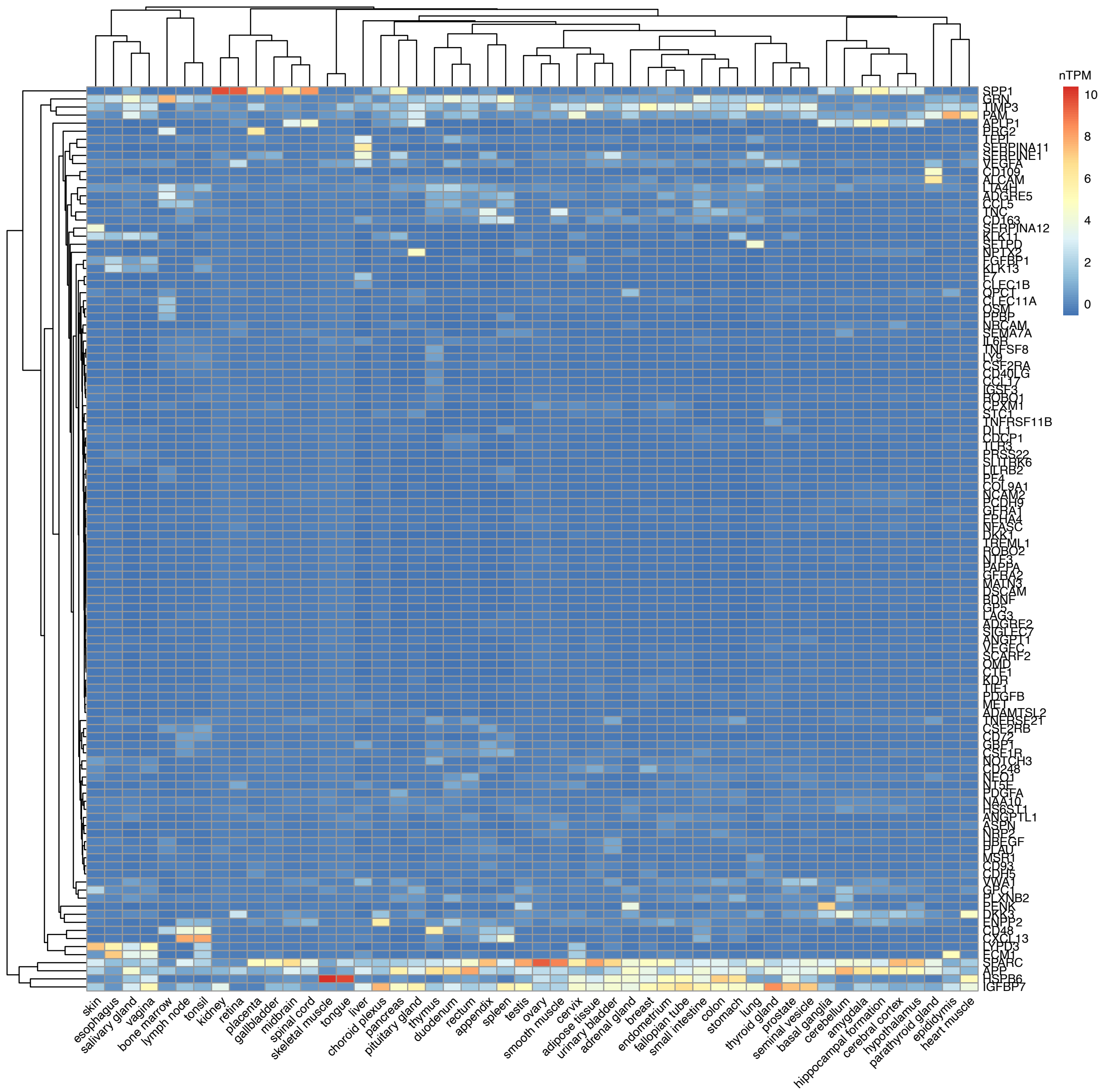

sFigure 18. Heatmap show expression levels of genes encoding proteins that showed consistent directional associations with plasma FLT1 abundance across BLSA and UKB. Expression levels across 50 tissue types were based on bulk RNA sequencing sourced from the Human Protein Atlas. Dendrograms reflect hierarchical clustering using Euclidean distances calculated from normalized Transcripts per Million (nTPM). nTPMs used to generate heatmaps were standardized within each gene to improve interpretability. Key: nTPM, normalized Transcripts per Million.

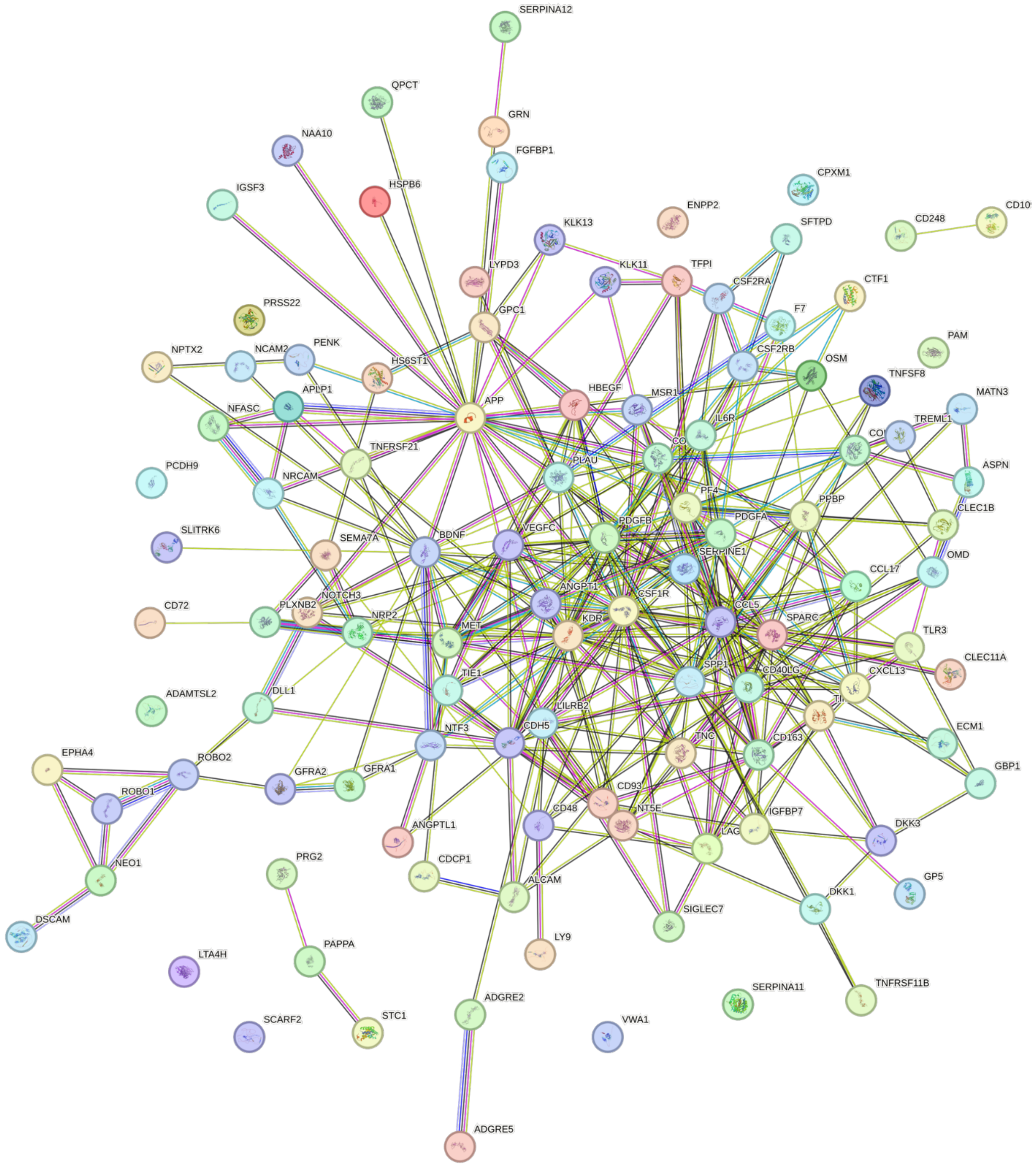

sFigure 19. Protein-protein interaction network of proteins that showed consistent directional associations with plasma FLT1 abundance across BLSA and UKB. Light blue lines indicate interactions derived from curated databases, and pink lines denote interactions that have been experimentally determined. Predicted interactions are shown using green lines for gene neighborhood, red lines for gene fusions, and blue lines for gene co-occurrence. Additional evidence channels include olive lines for text-mining support (co-mentioning in the scientific literature), black lines for co-expression evidence, and grey lines for protein homology. Results derived from STRING (Search Tool for the Retrieval of Interacting Genes/Proteins).

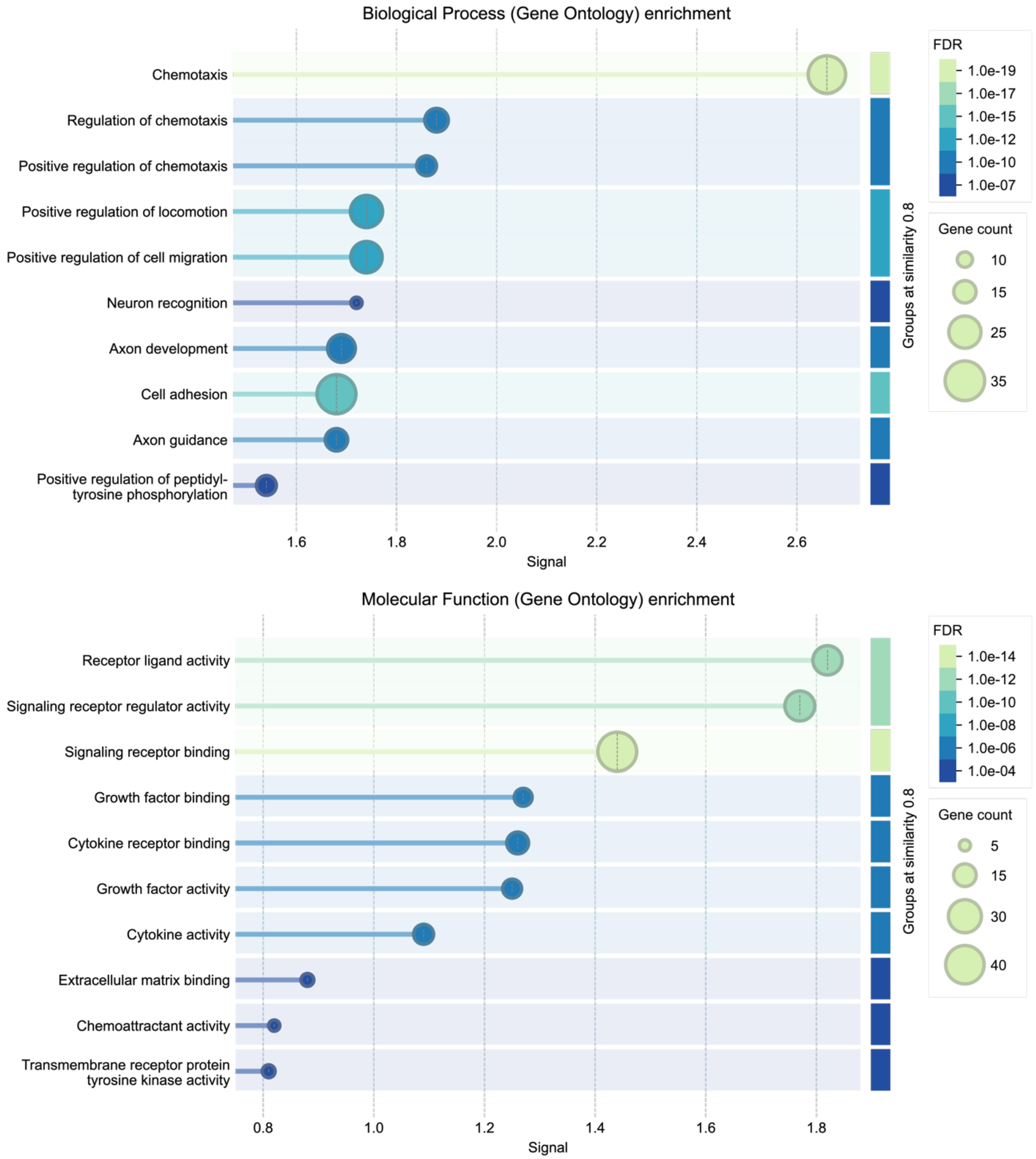

sFigure 20. Pathway enrichment (biological processes and molecular functions) of proteins that showed consistent directional associations with plasma FLT1 abundance across BLSA and UKB. Results derived from STRING (Search Tool for the Retrieval of Interacting Genes/Proteins).
